# Individual consistency in the learning abilities of honey bees: cognitive specialization within sensory and reinforcement modalities

**DOI:** 10.1007/s10071-022-01741-2

**Published:** 2023-01-06

**Authors:** Valerie Finke, Ricarda Scheiner, Martin Giurfa, Aurore Avarguès-Weber

**Affiliations:** 1 Zoologie II, Biozentrum, University of Würzburg, Am Hubland, 97074 Würzburg, Germany; 2 Centre de Recherches sur la Cognition Animale (CRCA), Centre de Biologie Intégrative (CBI), Université de Toulouse, CNRS, UPS, 118 Route de Narbonne, 31062 Toulouse, France; 3 Institut Universitaire de France, Paris, France

**Keywords:** Inter-individual variability, Insect cognition, Domain-general cognition, Domain-specific cognition, Cognitive repeatability, Honey bee

## Abstract

The question of whether individuals perform consistently across a variety of cognitive tasks is relevant for studies of comparative cognition. The honey bee (*Apis mellifera*) is an appropriate model to study cognitive consistency as its learning can be studied in multiple elemental and non-elemental learning tasks. We took advantage of this possibility and studied if the ability of honey bees to learn a simple discrimination correlates with their ability to solve two tasks of higher complexity, reversal learning and negative patterning. We performed four experiments in which we varied the sensory modality of the stimuli (visual or olfactory) and the type (Pavlovian or operant) and complexity (elemental or non-elemental) of conditioning to examine if stable correlated performances could be observed across experiments. Across all experiments, an individual’s proficiency to learn the simple discrimination task was positively and significantly correlated with performance in both reversal learning and negative patterning, while the performances in reversal learning and negative patterning were positively, yet not significantly correlated. These results suggest that correlated performances across learning paradigms represent a distinct cognitive characteristic of bees. Further research is necessary to examine if individual cognitive consistency can be found in other insect species as a common characteristic of insect brains.

**Supplementary Information:**

The online version contains supplementary material available at 10.1007/s10071-022-01741-2.

## Introduction

Cognition has been defined as the ability of animals to acquire, process, store and use vital information from the environment (Shettleworth 2009). While inter-individual differences in the cognitive skills in humans provide the basis of psychometrics, studies on animal cognition have generally neglected these differences in their attempt to underline the capacity of a given species to pass decisive cognitive tests (Boogert et al. 2018). Consequently, only the success of the most skilled individuals is usually highlighted. Alternatively, the average performance derived from individual data is used as a representative measure, leaving aside inter-individual differences, which could be informative about cognitive variation within a group (Pamir et al. 2011). Indeed, the existence of consistent inter-individual variability in cognitive traits is now well-studied across vertebrate species as it offers novel perspectives to study the link between cognition and behavioral syndromes or fitness (Matzel et al. 2003; Healy et al. 2009; Sih and Del Giudice 2012; Herrmann and Call 2012; Thornton et al. 2014; Guenther and Brust 2017; Dougherty and Guillette 2018; Cauchoix et al. 2018). These questions are relatively new in invertebrate research despite the tractability of these organisms for behavioral and neurobiological studies on inter-individual behavioral variability (Scheiner et al. 2005; Muller and Chittka 2012; Honegger and de Bivort 2018; Honegger et al. 2019; Tait et al. 2019; Tait and Naug 2020; Finke et al. 2021; Smith et al. 2022).

Social insects offer a great opportunity to study inter-individual cognitive differences due to their impressive cognitive capabilities (Dornhaus and Franks 2008; Avarguès-Weber et al. 2011; Giurfa 2013, 2019; Chittka 2017; Perry et al. 2017; Howard et al. 2018; Simons and Tibbetts 2019). Inter-individual variability has been described in a wide range of behaviors and is considered as a major factor for their ecological success, adding to division of labor and flexible responses to environmental changes (Thomson and Chittka 2001; Chittka and Muller 2009; Jeanson and Weidenmüller 2014; Bengston and Jandt 2014; Jandt and Gordon 2016; Walton and Toth 2016; Jeanson 2019). Variability in the learning abilities of bees has been connected to task allocation (Ray and Ferneyhough 1999; Ben-Shahar et al. 2000; Scheiner and Amdam 2009; Scheiner et al. 2017). For example, nectar and pollen foragers show inter-individual differences in their response thresholds to sucrose, correlating positively with differences in appetitive associative learning performances (Scheiner et al. 1999, 2001a, b; Pankiw and Page 1999). However, only a few studies have examined whether inter-individual differences in learning proficiency remain consistent over time and across different contexts. Given that some bees are better learners than others, do they have general learning skills making them better in multiple tasks and contexts, or are they rather specialized in a given set of problems? We previously showed that learning proficiency is stable over time in forager bees, justifying that their pattern of performances could be defined as a cognitive profile. We also evidenced that the performance in an elemental visual discrimination correlates positively with the performance in a non-elemental visual relational concept learning task where subjects have to follow a rule based on spatial relations between objects independently of the physical properties of those objects (Finke et al. 2021). By contrast, no clear correlation was observed between the learning performances in the olfactory and visual modality, suggesting that cognitive consistency is modality-specific (Finke et al. 2021). Cognitive specialization i.e. increased ability for a given cognitive trait relatively to other functions within individuals and by comparison to the general population, was also found when comparing elemental appetitive and aversive learning (Junca et al. 2019) and between landmark learning and olfactory learning (Tait et al. 2019). However, it remains to be determined if cognitive specialization in bees would mostly depend on the type of reinforcement used to train animals or if specialization depends on distinct “cognitive modules” sensu Fodor (1983), i.e. domain-specific and modality-dependent conglomerates with a fixed neuronal architecture, which could operate separately or in conjunction according to the complexity of the learning task.

**Fig. 1:**
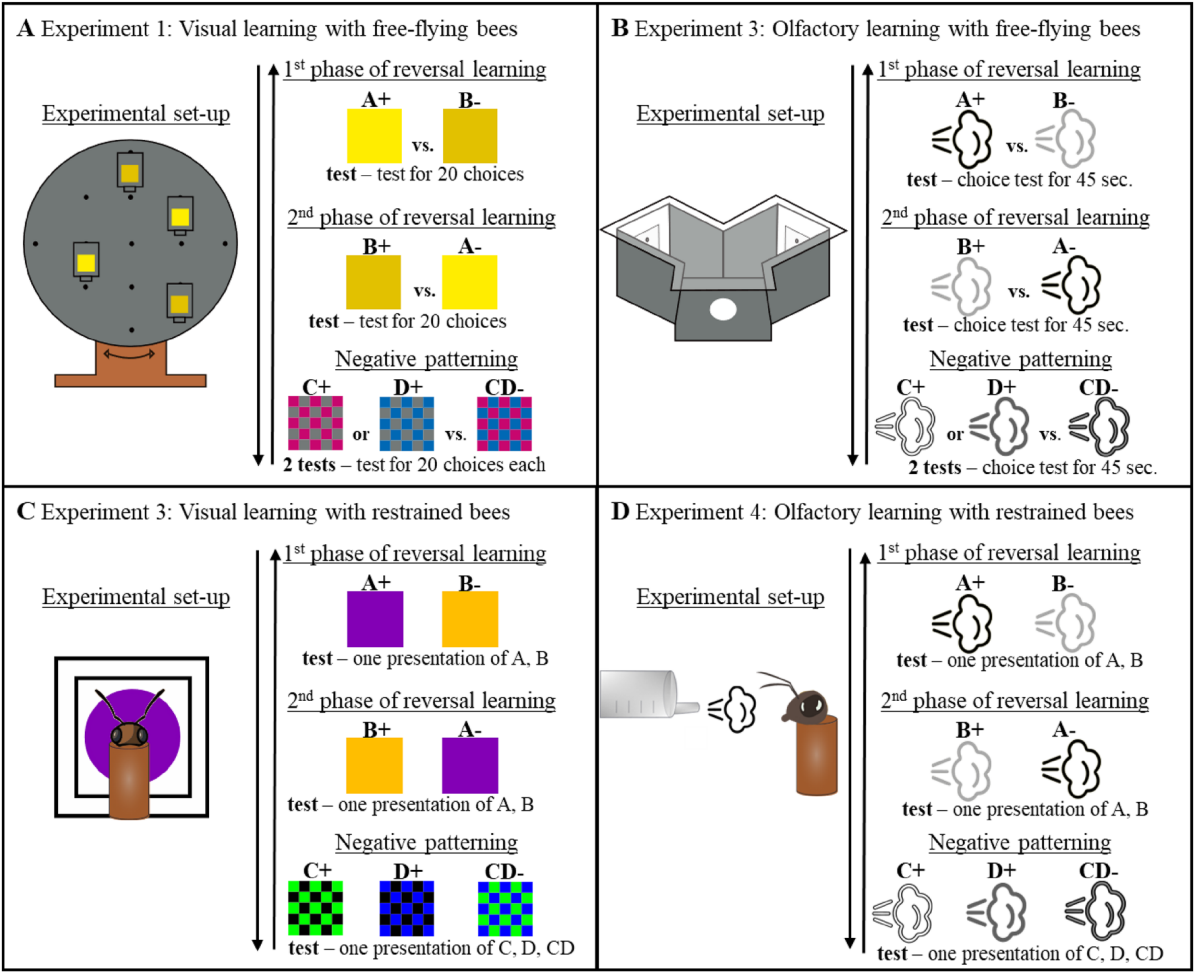
Schematic overview of the experiments conducted. **A** Experiment 1: Visual learning with free-flying bees. The experimental set-up was a rotating screen apparatus where hangers could be attached to at various locations. The hangers displayed the stimuli during conditioning and testing. For reversal learning we used yellow and greenish-yellow cardboard squares as visual stimuli. For negative patterning we used checkerboard squares cut from pink or blue cardboards to create the single stimuli C/D and blue and pink cardboards to create the compound stimulus CD. Test performances were assessed by a 45 s. choice tests during which all contacts of the bees with the respective stimuli were counted and a percentage of correct choices was then calculated for each test. **B** Experiment 2: Olfactory learning with free-flying bees. The experimental set-up was a Y-maze apparatus where the bees could fly through a hole to enter and get access to the inside where the olfactory stimuli were applied to filter papers on the backwalls of the maze. In reversal learning we used the odors linalool and 2-hexanone as A and B. For negative patterning we used limonene as stimulus C, 2-octanol as stimulus D and a mixture of these odors as CD. Test performances were assessed by a 20 choice tests during which all contacts of the bees with the respective stimuli were counted and a percentage of correct choices was then calculated for each test. **C** Experiment 3: Visual learning with restrained bees. The experimental set-up was a box with five chambers covered by movable red Plexiglas^®^ ceilings preventing light stimulation. In reversal learning we used 400 nm and 600 nm monochromatic light discs as stimulus A and B. For negative patterning we used a blue or green checkerboard as single stimuli C/D and a blue-green checkerboard as compound stimulus CD. Test performances were assessed by a single presentation of each stimulus of the respective learning task. **D** Experiment 4: Olfactory learning experiments with restrained bees. The experimental set-up was also a movable box with compartments for ten bees in front of an exhaust fan. Odors were delivered through an airstream. In reversal learning we used the odors linalool and 2-hexanone as A and B. For negative patterning we used limonene as stimulus C, 2-octanol as stimulus D and a mixture of these odors as CD. Test performances were assessed by a single presentation of each stimulus of the respective learning task (color figure online)

Here we aimed at testing whether individual performances correlate between different learning tasks relying on the same reinforcement and sensory modality or whether distinct cognitive modules mediate performance in these tasks. We tested bees in (i) a reversal learning task (Pavlov 1927) in which subjects are trained to discriminate a rewarded and a non-rewarded stimulus in two consecutive phases with a change of reward contingencies between phases (A+ vs. B− and then A− vs. B+), and (ii) a negative patterning discrimination in which subjects have to learn to respond to the presentation of single reinforced stimuli but not to their conjunctive presentation (e.g. C+ and D+ vs. CD−). Reversal learning has the advantage of using the first phase (A+ vs. B−) as a proxy for the animals’ capacity to solve an elemental discrimination, and the second phase as a proxy of their flexibility to reverse this discrimination (Ben-Shahar et al. 2000; Hadar and Menzel 2010; Mota and Giurfa 2010; Boitard et al. 2015). The second phase induces indeed a transient stimulus ambiguity in terms of learned valence that needs to be overcome. Importantly, only individuals that learned the initial A+ B− association in the 1st phase of reversal learning can be evaluated for their ability to reverse this association in the 2nd phase (Mota and Giurfa 2010). The negative patterning discrimination (Whitlow and Wagner 1972) can only be solved if the compound stimulus is treated as being different from the sum of its components, which requires inhibiting stimulus summation and implementing different forms of processing such as configural processing (Deisig et al. 2001; Schubert et al. 2002; Devaud et al. 2015). Both learning paradigms were conducted using stimuli from different sensory modalities (visual or olfactory) and different set-ups involving conditioning protocols that involved either classical or operant type of conditioning to examine whether the patterns of individual consistency in performance between tasks are stable across these different contexts. In a series of four experiments (see Fig. 1 for an overview of the experiments), we tested bees consecutively in the two learning tasks explained in detail above. Two experiments with free-flying bees involved both a combination of operant (flying to the correct target) and classical (association between the CS and the reinforcement) learning either in the visual (experiment 1) or in the olfactory modality (experiment 2). The remaining two experiments involved pure classical conditioning of restrained bees and consisted of conditioning their proboscis extension reflex (PER) with either visual (experiment 3) or olfactory stimuli (experiment 4). Experiments on PER conditioning with restrained bees in the laboratory have the great advantage of providing standardized external (e.g., temperature and humidity) and experimental conditions (e.g., timing and duration of trials, stimuli illumination and concentrations). Additionally, they allow testing of multiple bees per day and enable therefore the access to large sample sizes. However, they provide us only with restricted information of individuality in the test performances as performance is quantified as a binomial variable (response or no response), thus precluding fine-grain analyses of performance. Experiments with free-flying bees have the advantage of providing a more detailed grain analyses of individual data of test performances as learning can be quantified by the percentage of correct choices reached by each individual. On the contrary, these experiments require considerable time and focus on single individuals and restrict, in comparison, the sample sizes considerably. With such a portfolio of experiments differing in both procedure and sensory modality we aimed determining if individuals exhibit across-task consistency in their cognitive success, thus indicating the presence of abilities that would be independent of a specific experimental context. Our hypothesis was that within each experiment performances would correlate positively across all tasks and that we would find similar patterns of correlations in performances across the different experiments. We hypothesized this based on our recent findings showing that learning proficiency differs between individuals but remains consistent over time and across an elemental and an alternative non-elemental learning task as long as the stimuli were from the same sensory modality (Finke et al. 2021).

## Experiment 1: visual learning in free-flying bees

### Material and methods

#### General methods

The experiments were conducted within an indoor flight cage (~ 4 × 6 m) hosting a single colony. The flight cage was made from UV-transparent Plexiglas, thus providing light conditions that were similar to natural daylight conditions. The bees were provided with pollen ad libitum, a water source and an artificial gravity feeder containing sucrose solution (30% weight/weight). The bees used for the experiment were recruited from the feeder to the experimental set-up. The set-up consisted of a vertically mounted rotatable grey plastic screen (rotating screen; 50 cm in diameter), where hangers (6 × 8 cm) could be attached at various locations (see Fig. S1 for a schematic overview of the apparatus). The apparatus was achromatic for the bees. The hangers allowed to display stimuli (5 × 5 cm) and had a landing platform where the bees could land on to collect 10 µl of the reward (50% sucrose solution, weight/weight), punishment (quinine solution, 60 mM) or water. A punishment is commonly used in discrimination learning protocols to improve visual stimulus differentiation (Avarguès-Weber et al. 2010). In the case of the negative patterning paradigm, as the compound stimulus opposed to the single rewarded stimuli should not be reinforced, we provided water as neutral US stimulus (Deisig et al. 2001; Schubert et al. 2002). Bees (*n* = 33) were first pre-trained to collect ad libitum sucrose solution from the landing platforms of two hangers (see Fig. S1) in the absence of stimuli until they landed quickly after arriving at the set-up for at least five times. Once bees completed this pre-training, the learning protocols were initiated.

During training and testing only one bee, individually marked with a colored spot on the thorax (Uni-posca paint marker; Mitsubishi Pencil Co., Ltd.), was present at a time at the experimental set-up. Other bees approaching the setup were captured into cages to avoid them interfering with the focal bee. The total number of trials varied according to the learning tasks and are specified below in each case. During each trial, a choice for a given stimulus was recorded once the bee had landed on a platform and tasted the corresponding solution. If the bee made in incorrect choice, it was allowed to make further choices until a correct choice was scored. Having made a correct choice, the bee was transferred to a plexiglas spoon providing a sucrose solution, which was then moved 1 m away from the screen, while the screen was rotated to change the spatial positions of the stimuli. Subsequently, the solutions on the hangers were refilled. The bee was then allowed to make another choice. Bees usually made 3–5 choices per foraging bout. When they returned to the hive, the hangers were cleaned with 50% ethanol and all solutions were refilled.

Each acquisition phase was directly followed by a non-reinforced test in which the bee had to choose between the trained stimuli. Each stimulus was presented twice as it occupied two hangers. Fresh stimuli and hangers were used during the test. After 20 choices a test was finished. A choice was defined as either landing or touching the landing platform or test stimulus. Half of the bees were first subjected to the reversal learning task and then to the negative patterning task while the task order was reversed for the other half. Once a learning paradigm was completed, the bee was allowed to collect sucrose solution on the hangers in the absence of any stimulus for three foraging bouts before the second learning paradigm started. Only highly motivated bees coming back to the experimental set-up regularly (with a maximum of 10 min between visits, usually 2–5 min) were kept for analysis. The whole procedure took 6–8 h per bee.

#### Reversal learning protocol

In the first phase of reversal learning one color was associated with a reward (A+) while a second color was associated with a punishment (B−). In the 2nd phase, the reward contingencies were reversed, so that the previously rewarded target stimulus became punished and vice versa (A− and B+). Each stimulus was presented on two hangers so that four hangers were presented during conditioning trials and in the tests. Both phases amounted to 30 trials in total and each phase was directly followed by a non-rewarded test presenting A and B in the absence of reinforcement. Colors used were squares cut from HKS-3N and HKS-68N cardboard (5 × 5 cm; HKS-N pigment papers; Hostmann-Steinberg K + E Druckfarben, H. Schmincke and Co., Germany) that appear yellow and greenish-yellow to the human eye (see Fig. S2 for the spectral reflectance curves of the stimuli and their positions in the hexagon color space, a model for color perception of bees, Chittka 1992). Half of the bees were initially conditioned with HKS-3N as stimulus A and HKS-68N as stimulus B while the other half experienced a reversed stimulus contingency. Color loci in the hexagon were separated by 0.07 hexagon units, which is sufficient to be discriminated by the bees (Chittka 1992; Dyer and Neumeyer 2005; Avarguès-Weber et al. 2010). During acquisition and testing two correct and incorrect colored squares were displayed at the same time in varying positions and dispositions on the rotating screen.

#### Negative patterning protocol

The acquisition phase consisted of three different consecutive blocks of trials: Two blocks consisted of presenting at each trial four hangers displaying only one of the rewarded single stimuli (C+ or D+), a third block consisted of presenting the non-reinforced compound stimulus (CD−) on two hangers and a rewarding black-and-white checkerboard alternative (XY+) on two hangers. The addition of the checkerboard is necessary in the case of experiments with free-flying bees as presenting only the compound stimulus (CD−) in consecutive non-reinforced trials would result in a decrease of motivation and in the bees ceasing their foraging activities at the set-up. Presenting a rewarded neutral alternative (XY+) allows overcoming this problem while keeping the ambiguity of stimulus valence for C and D. This alternative was used successfully by Schubert et al. (2002) to study negative patterning in free-flying bees. The order of blocks throughout acquisition was pseudo-randomized so that each block was not conducted more than twice in a row. Each block lasted for one foraging bout, as stimuli were exchanged once a bee returned to the hive. Consequently, the number of trials in each block varied. Usually, the C+ and D+ blocks amounted to 3–6 trials and the CD− blocks to 4–8 trials per foraging bout. The experiment was completed when the bee reached 30 trials for both the C+ and D+ blocks and 60 trials for the CD− block, i.e. 120 trials in total. In this way, each bee experienced 60 rewarded and 60 non-rewarded experiences. Again, for each trial a choice was recorded once the bee landed on a platform and tasted the corresponding solution. The acquisition phase was followed by two non-rewarded tests where CD− and either C+ or D+, respectively, were presented together on two hangers each. None of the test stimuli provided reinforcement. The tests were completed when the bee performed 20 choices in total. The two tests were spaced by one refreshing foraging bout in which the reinforced trained stimuli were offered to maintain a high motivation. The square stimuli were cut from HKS-26N, HKS-44N, HKS-92N and HKS-88N cardboards (5 × 5 cm; HKS-N pigment papers; Hostmann-Steinberg K + E Druckfarben, H. Schmincke and Co., Germany) and appeared pink, blue, grey and black to the human eye, respectively (see Fig. S2 for the spectral reflectance curves of the stimuli and their positions in the hexagon color space). The pink (26N) and blue stimuli (44N) were separated by 0.07 hexagon units, which is a perceptual distance sufficient to support discrimination (Chittka 1992; Dyer and Neumeyer 2005; Avarguès-Weber et al. 2010). The two elemental stimuli (C and D) consisted of checkerboard patterns made of 1 × 1 cm squares of either the pink or blue cardboard on the HKS-92N background. The compound stimulus (CD) was thus a checkerboard pattern made of the pink and blue cardboards. The rewarding alternative (XY) was a black and white checkerboard made of squares of the same size (i.e. 1 × 1 cm each). This design was adapted from (Schubert et al. 2002).

### Statistical analysis

Test data were used to assess the individuals’ learning performances as we did not observe, at the individual level, a sigmoidal increase of performance in the acquisition starting at a 50% random choice level and increasing significantly as classically observed at the group level. This is probably due to the stochasticity of choices, as bees have a 50% probability of making a correct choice at each trial, which may result by chance in unexpected high or low scores at the individual level (see Figs. S5 for analysis of the acquisition and test phases at the group level). We thus used the percentage of correct choices in the non-reinforced tests to assess individual consistency across the three leaning tasks tested.

Individual consistency across the different tasks was tested using Spearman rank correlations between test performances. Reversal learning ability can only be tested on individuals that successfully acquired the first A+B− association (Mota and Giurfa 2010). Most bees chose preferentially A in the test following the first phase and were consequently kept for analysis of their reversal learning ability (*n* = 27 of 33). We decided to use an arbitrary threshold of 60% correct choices in the test to consider a bee as learner or non-learner. To assess whether the order in which the learning tasks were conducted, or which stimulus was rewarded in reversal learning influenced test performances generalized linear mixed models (GLMM) were used. The models with a binomial error structure and logit-link function included the choices made (either correct scored as 1 or incorrect scored as 0) in the test as dependent variable and the order of the tasks (*order*) and the rewarded stimulus (*group_RL*) as fixed factors. The bees’ identity (*subject*) was included as a random factor. Different models were performed where the factors were gradually removed and compared using an ANOVA. P-values from these comparisons were provided to account for each factor impact. The model with the lowest AIC value was chosen as most appropriate fit (S10-S12).

All GLMMs were performed using R Statistical Software version 3.6.3 (R Core Team 2022) with the package lme4 (Bates et al. 2015). All other statistical analyses and graphs were performed using GraphPad prism version 9.0.0 (GraphPad Software Inc., San Diego, California, USA). The significance level was *α* = 0.05 to account for our relatively small sample sizes (Lakens et al. 2018). For all correlations of the test performances across the three learning tasks, the null hypothesis was that the correlation coefficient rho was not different from zero.

### Results

We analyzed whether individual performances of free-flying bees (*n* = 33) in a reversal learning problem correlated with performances in a negative-patterning problem, both established using visual stimuli. As reversal learning consists of two phases (A+ vs. B− → A− vs. B+), we performed separated analyses between negative patterning performances and performances in the 1st and 2nd phases of the reversal learning protocol. Only bees that successfully learned the initial discrimination of the reversal learning (≥ 60% correct choices in the test) were used for correlations including the 2nd reversal learning phase as successful reversal learning requires learning of the initial discrimination. Including bees that did not learn in the first phase goes against the definition of reversal learning as these bees did not have to overcome the transient stimulus ambiguity that characterizes the transition between phases. Importantly, neither the order in which reversal learning and negative patterning were conducted (*order)*, nor the stimulus which was rewarded in reversal learning (*group_RL)* affected test performances (GLMM: Order; *n* = 33, **1st RL**: ${\chi }_{(1)}^{ 2}$= 0.32, *p* = 0.57, **2nd RL**: ${\chi }_{(1)}^{ 2}$ = 0.04, *p* = 0.85, **NP**: ${\chi }_{(1)}^{ 2}$ = 2.96, *p* = 0.09; group_RL: *n* = 33, **1st RL**: ${\chi }_{(1)}^{ 2}$= 2.1, *p* = 0.08, **2nd RL**: ${\chi }_{(1)}^{ 2}$ = 1.41, *p* = 0.24, **NP**: ${\chi }_{(1)}^{ 2}$ = 0.15, *p* = 0.70; tables S4–S6 in the supplementary).

**Fig. 2:**
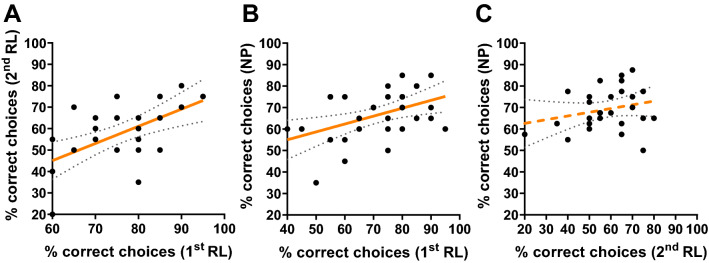
Correlations of individual test performances in Experiment 1: Visual learning experiment with free-flying bees. Pairwise Spearman rank correlations between the test performances (Percent of correct choices) of individual bees in **A** the 1st phase of reversal learning (1st RL) and the 2nd phase of reversal learning (2nd RL; *n* = 27, rho = 0.53, *p* = 0.005, *R*^2^ = 0.34), **B** the 1st phase of reversal learning and negative patterning (NP; *n* = 33, rho = 0.42, *p* = 0.02, *R*^2^ = 0.2) and **C** the 2nd phase of reversal learning and negative patterning (*n* = 27, rho = 0.25, *p* = 0.201, *R*^2^ = 0.06). Each dot represents data from one bee. The regression line is indicated in orange and the dotted grey lines show the 95%-confidence intervals of the regression. Solid regression lines indicate a significant correlation and dashed lines indicate a non-significant correlation (color figure online)

Figure 2 shows that individual learning performances in the 1st discrimination phase of reversal learning correlated with performances in the 2nd phase of reversal learning (Spearman rank correlation, *n* = 27, rho = 0.53, *p* = 0.005, *R*^2^ = 0.34, Fig. 2A, Table 1). Most bees (*n* = 4 out of 6 non-learner bees in the 1st phase) that were excluded because they were considered as non-learners in the 1st phase also performed around a 50% chance level (ranging between 40 and 55% correct choices) in the 2nd phase of reversal learning. Two non-learner bees performed well (70 and 80% correct choices) in the 2nd phase of reversal learning but this cannot be seen as a case of reversal learning (see above) but rather as an elemental learning performance given that they did not learn to reverse the reinforcement contingency. Individual test performances were also positively correlated between the 1st phase of reversal learning and in the negative patterning procedure (*n* = 33, rho = 0.42, *p* = 0.02, *R*^2^ = 0.2, Fig. 2B, Table 1). However, no significant correlation was observed between the 2nd phase of reversal learning and negative patterning (*n* = 27, rho = 0.25, *p* = 0.201, *R*^2^ = 0.06, Fig. 2C, Table 1). Here, most bees (*n* = 4 out of 6) that were non-learners in the 2nd phase of reversal learning succeeded nevertheless in the negative patterning discrimination.

**Table 1:** Results of the Spearman rank correlations comparing the individual’s test performances in the 1st phase of reversal learning (1st RL), the 2nd phase of reversal learning (2nd RL) and negative patterning (NP)

Experiment	Correlation	Rho	*P* value
1	1st RL–2nd RL	0.53	**
1	1st RL–NP	0.42	*
1	2nd RL–NP	0.25	ns
2	1st RL–2nd RL	0.60	***
2	1st RL–NP	0.46	*
2	2nd RL–NP	0.19	ns
3	1st RL–2nd RL	–	–
3	1st RL–NP	0.18	*
3	2nd RL–NP	0.18	ns
4	1st RL–2nd RL	–	–
4	1st RL–NP	0.33	**
4	2nd RL–NP	0.15	ns

*ns* not significant

*p < 0.05, **p < 0.01, ***p < 0.001

## Experiment 2. olfactory learning in free-flying bees

### Material and methods

#### General methods

We used a Y-maze apparatus placed on the garden of our apiary and protected by an umbrella from direct sunlight (see Fig. S3 for a schematic overview of the apparatus). The maze was illuminated by natural daylight and was composed of a sliding door allowing to control the exclusive access of a focal bee, an entrance arm leading to a decision chamber via a small aperture (6 cm in diameter) where the bees could choose between the two arms presenting the olfactory stimuli (arms dimensions: length: 40 cm, height: 20 cm, width: 20 cm). The backwalls (20 × 20 cm) of the two arms were placed at a distance from 15 cm to the decision chamber and coated with white copy paper. The odorant stimuli were applied onto a filter paper (5 × 5 cm) taped to a cardboard which was attached to the copy paper coating the backwalls. The whole Y-maze was covered by movable UV-transparent Plexiglas elements.

Bees (*n* = 22), marked individually with paint marker (Uni-posca paint marker; Mitsubishi Pencil Co., Ltd.) were recruited at a gravity feeder and pre-trained in a stepwise fashion to enter the Y-maze, fly through the entrance hole to access the decision chamber and collect a reward of sucrose solution from the backwalls where no odor stimulus was presented. Only one individual was trained and tested at a time. During acquisition phases, one odorant was rewarded with sucrose solution (50%, weight/weight) while a different odorant was punished with quinine solution (60 mM; for reversal learning) or associated with water (for negative patterning). Odorants were presented on a 5 × 5 cm filter paper onto which 10 µl of a pure odorant were applied. The odorant paper was attached with tape to the copy paper covering the maze backwalls. Reinforcement was delivered by means of transparent micropipettes tips located in the center of each backwall and odorant paper. The solution was not contaminated by the odors, as they were directly filled into the micropipette tips. The side of the rewarded stimulus was changed in a pseudo-random sequence to prevent positional learning. In each acquisition trial, bees were required to enter the Y-maze, fly to the decision chamber and choose between odorants displayed at the two backwalls of the Y-maze. A correct choice led to an ad libitum reward of sucrose solution and an incorrect choice led to the tasting of quinine/water. In case of an incorrect choice, bees were allowed to collect subsequently sucrose solution from the alternative arm displaying the correct stimulus. Within each trial only the first choice of bees was recorded. A choice was scored once bees crossed an imaginary line that was 5 cm distant from the backwalls, i.e. from the odor stimuli. As bees received an ad libitum reward upon each correct choice, one trial amounted to one foraging bout. Between trials the Y-maze was cleaned with 50% ethanol and ventilated so that potentially remaining odors were removed. Then the paper cover of the backwalls was exchanged and fresh odorants were applied. Acquisition phases were immediately followed by non-reinforced tests with fresh stimuli. Each test was conducted twice to swap stimulus sides and lasted 45 s. During this period, all contacts with the stimuli were recorded. Between tests, bees were subjected to three reinforced conditioning trials (‘refreshing trials’) to maintain a high appetitive motivation. Half of the bees were first subjected to reversal learning and the other half to negative patterning. Each learning protocol was spaced by three foraging bouts where bees could collect sucrose solution at the entrance of the maze without any stimuli present. Only motivated foragers which completed both tasks and returned quickly to the experimental set-up (< 10 min, usually 2–5 min) were kept for analyses. The whole procedure took around 6 to 8 h per bee.

#### Reversal learning protocol

In the 1st phase of reversal learning, bees had to distinguish between two odorants, linalool and 2-hexanone (Sigma-Aldrich Chemie GmbH), one being associated with a reward of sucrose solution (A+) while the second odorant was associated with a punishment of quinine solution (B−). The odorants could be easily discriminated by bees (Laska et al. 1999). Half of the bees were trained with linalool as stimulus A and hexanone as stimulus B while odor identity was exchanged for the other half. Then in the 2nd phase the reward contingencies of the previous phase were reversed (A− vs. B+). Each phase amounted to 10 trials i.e. foraging bouts. Each acquisition phase was directly followed by two unreinforced retention tests (where the side of the stimuli were swapped between tests) presenting fresh stimuli.

#### Negative patterning protocol

The acquisition phase consisted of three types of trials presented in a pseudo-random order: C+ and D+ trials presented these rewarded odorants in both arms of the Y-maze. CD− trials offered the non-rewarded CD− compound vs. a rewarding alternative odorant X+ , which was used to keep the bees coming to the setup (see above). Limonene was used as C+ , 2-octanol as D+ and Nonanal a X+ . All odorants were obtained from Sigma-Aldrich Chemie GmbH. These odorants can all be well discriminated by the bees (Laska et al. 1999). The whole acquisition consisted of 20 trials, including 5 C+ , 5 D+ and 10 CD-/X+ trials. After the acquisition phase, two non-reinforced tests were conducted, both in the absence of reinforcement, one presenting C vs. CD and the second presenting D vs. CD.

### Statistical analysis

As in experiment 1 test data were used to assess the individuals’ learning performances as we did not observe, at the individual level, a sigmoidal increase of performance in the acquisition starting at a 50% random choice level and increasing significantly as classically observed at the group level (see Fig S6 for analysis of the acquisition and test phases at the group level). We thus used the percentage of correct choices in the non-reinforced tests to assess individual consistency across the three leaning tasks tested.

Individual consistency across the different tasks was tested using Spearman rank correlations between test performances. Reversal learning ability can only be tested on individuals that successfully acquired the first A+B− association (Mota and Giurfa 2010). In both experiments, most bees chose preferentially A in the test following the first phase and were consequently kept for analysis of their reversal learning ability (*n* = 20 of 22). We decided to use an arbitrary threshold of 60% correct choices in the test to consider a bee as learner or non-learner. To assess whether the order in which the learning tasks were conducted or which stimulus was rewarded in reversal learning influenced test performances generalized linear mixed models (GLMM) were used (see the statistical analysis paragraph of experiment 1 for a detailed description of the GLMMs and model selection procedure, Tables S10–S12).

## Results

We analyzed if individual performances of free-flying bees (*n* = 22) in a reversal learning discrimination correlated with performances in a negative-patterning problem, both established using olfactory stimuli. As before, we performed separated analyses between negative-patterning performances and performances in the 1st and 2nd phases of reversal learning. Only bees that successfully learned the initial discrimination of the reversal learning (≥ 60% correct choices in the test) were used for correlations including the 2nd reversal learning phase as successful reversal learning requires learning of the initial discrimination. There were no significant effects of the sequence of problems trained (*order*) or of the stimulus which was rewarded in reversal learning (*group_RL*) on test performances (GLMM: *Order*: *n* = 22, **1**^**st**^** RL:** ${\chi }_{(1)}^{ 2}$ = 2.03, *p* = 0.15, **2**^**nd**^** RL:** ${\chi }_{(1)}^{ 2}$ = 0.05, *p* = 0.85, **NP:** ${\chi }_{(1)}^{ 2}$ = 0.04, *p* = 0.82, Tables S10–12; group_RL: *n* = 22 **1**^**st**^** RL:** ${\chi }_{(1)}^{ 2}$ = 0.17, *p* = 0.68, **2**^**nd**^** RL:** ${\chi }_{(1)}^{ 2}$ = 0.35, *p* = 0.55, **NP:** ${\chi }_{(1)}^{ 2}$ = 1, *p* = 0.32, Tables S10–S12).

**Fig. 3:**
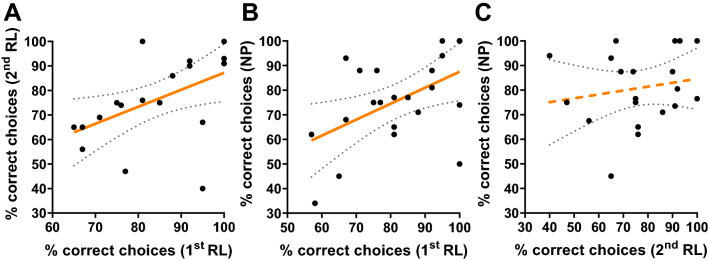
Correlations of the individual test performances in Experiment 2: Olfactory learning experiment with free-flying bees. Pairwise Spearman rank correlations between test performances (Percent of correct choices) of individual bees in **A** the 1st phase of reversal learning (1st RL) and the 2nd phase of reversal learning (2nd RL; *n* = 20, rho = 0.6, *p* = 0.006, *R*^2^ = 0.25), **B** the 1st phase of reversal learning and negative patterning (NP; *n* = 22, rho = 0.46, *p* = 0.03, *R*^2^ = 0.25) and **C** the 2nd phase of reversal learning and negative patterning (*n* = 20, rho = 0.19, *p* = 0.41, *R*^2^ = 0.03). Each dot represents the data of one bee. A regression line is indicated in orange and the dotted lines show the 95%-confidence intervals of the regression. Solid regression lines indicate a significant correlation and dashed lines indicate a non-significant correlation (color figure online)

Figure 3 shows that test performances remained consistent across the two phases of reversal learning (Spearman rank correlation, *n* = 20, rho = 0.6, *p* = 0.006, *R*^2^ = 0.25, Fig. 3A, Table 1). The two bees that were excluded from this analysis, as they did not learn the initial discrimination, also did not show any sign of learning in the 2nd phase of reversal learning (38% and 50% correct choices).Test performances also remained consistent across the 1st phase of reversal learning and negative patterning (*n* = 22, rho = 0.46, *p* = 0.03, *R*^2^ = 0.25, Fig. 3B, Table 1). As for the visual modality, no significant correlation was found between test performances of the 2nd phase of reversal learning phase and negative patterning (*n* = 20, rho = 0.19, *p* = 0.41, *R*^2^ = 0.03, Fig. 3C, Table 1). Here one excluded bee also failed in negative patterning while the other just reached the learner threshold (62% correct choices).

## Experiment 3. visual learning in restrained bees

### Material and methods

#### General methods

The day before the learning experiments, returning non-pollen foragers from a single colony were individually caught in glass vials at the hive entrance. The vials were placed on crushed ice until the bees ceased their movements. They were then harnessed in plastic tubes with their heads fixed by two metal pins, allowing only minimal movements (Dobrin and Fahrbach 2012; Mancini et al. 2018). Thirty minutes after fixation, the bees were fed with 10 µl of sucrose solution (30% weight/weight) and stored in a dark and humid box at room temperature (~ 25 °C) for approximately 15 h. Experiments were performed in a dark room under weak red-light illumination, invisible for the bees, using the set-up described in detail by (Mancini et al. 2018). The experimental set-up consisted of a box with five chambers (10 × 10 × 10 cm) covered by movable red Plexiglas® ceilings preventing light stimulation between trials. In each chamber a bee was positioned vertically at 4 cm distance in front of a tracing paper screen (10 × 10 cm) onto which the visual stimuli were projected. Conditioned stimuli were different between protocols and are described below.

Bees (*n* = 140) were first tested for intact PER by stimulating the antennae with a sucrose solution (50% weight/weight). Only bees that fully extended their proboscis, i.e. showing high motivation for the reinforcement (Scheiner et al. 1999, 2005), were included in the experiments. Thirty minutes prior to the start of the experiment the bees were placed in the conditioning chambers to habituate to the set-up. Both learning protocols followed the same standardized protocol. Ten bees were conditioned in “parallel”, i.e. they completed one trial, one after the other. Each trial lasted 30 s and the inter-trial interval was five minutes. In rewarded trials, the stimulus was presented for 16 s and a 50% sucrose solution was delivered 14 s after onset of stimulus presentation with two seconds overlap and two seconds of reward alone. In unrewarded trials the stimulus was presented for 16 s in the absence of reward. For the remaining 12–14 s of each trial the bee remained in its position without stimulation. Sucrose was delivered by touching the bees’ antennae with a toothpick soaked in the sucrose solution to trigger the PER and allowing then the licking of the solution with their proboscis. Before US onset, the toothpick was always kept outside of the chamber to avoid responses to water vapor (Kuwabara 1957). Once the acquisition phase was completed, non-reinforced tests were conducted five minutes after the last trial. During tests, each trained stimulus was presented once during 16 s without reinforcement. The order in which the stimuli were presented during acquisition and in the test was pseudo-randomized. For each acquisition and test trial the conditioned response to the colors (i.e. extension of the proboscis; 1 = response, 0 = no response) was recorded only during the 14 s of visual stimulation alone. Importantly, a response was scored differently compared to most studies on olfactory PER conditioning. Usually, a response is scored if the proboscis extends beyond a virtual line between the open mandibles (Deisig et al. 2002, 2003; Komischke et al. 2005; Matsumoto et al. 2012). However, such a strong response to the visual stimuli was almost never observed in pilot experiments (< 10%) even though the bees responded to the sucrose solution with a full PER. In consequence, a PER was scored as positive once the proboscis extended by 45° from its resting position, so once the proboscis reached a virtual line between the open mandibles. The reasons for this difference in PER strength to visual and olfactory stimuli are unclear but it may reflect the capacity and pertinence of PER to reflect learning for stimuli of both modalities. While visual information may guide distantly the bees’ approach to a visual target, odorants might be more relevant at a closer range, for instance upon landing, and may thus act as triggers of proboscis extension.

After the retention test, PER integrity was checked again and bees that did not respond were discarded from the analyses (< 5%). Additionally, all bees that did not respond to the sucrose stimulation in any conditioning trial were also discarded. The bees were subjected to both learning protocols (reversal learning and negative patterning) on the same day spaced by one hour resting time to ensure a high appetitive motivation. Only bees that completed both tasks were kept for analysis. The order in which the bees were subjected to each protocol was randomized across test days. Conditioning and testing took 7.5 h for 10 bees.

#### Reversal learning protocol

In the 1st phase of reversal learning, one visual stimulus (A+) was associated with a sucrose reward while another visual stimulus was not reinforced (B−). The visual stimuli were colored discs (3 cm diameter) projected onto the tracing paper screen of the conditioning chamber via an optic fiber connected to a monochromator (Polychrome V^®^, Till Photonics, Germany). A custom-made software controlled the stimuli wavelengths, their intensity, the onset- and off-set of visual stimuli, and the inter-trial interval. Each colored disc subtended a visual angle of 40° to the bees’ eye, ensuring perception of the chromatic properties of the stimuli (Giurfa et al. 1996, 1997; Mancini et al. 2018). The stimuli were monochromatic lights peaking at either 400 or 600 nm and appeared violet and orange to the human’s eye respectively (see Fig. S4 for the spectral reflectance curves of the stimuli and their positions in the hexagon color space). The colors of the stimuli were separated by 0.67 hexagon units which is sufficient to be discriminated by the bees (Chittka 1992; Dyer and Neumeyer 2005; Avarguès-Weber et al. 2010). Half of the bees were trained with violet as stimulus A and orange as stimulus B, while the other half had color identity reversed. Once the 1st phase was completed, the bees remained in the experimental set-up for 30 min before the start of the 2nd phase. In this phase, the previously rewarded stimulus became unreinforced (A−) and the unreinforced stimulus became associated with a reward (B+). Both acquisition phases consisted of 16 trials in total, with the rewarded and the unrewarded stimuli presented eight times each in a pseudo-random sequence. Each acquisition phase was followed by two consecutive non-reinforced tests, each presenting once one of the two training stimuli.

#### Negative patterning protocol

The acquisition phase consisted of 32 trials which were divided into eight blocks of four trials. Each block contained one presentation of each of the two elemental stimuli (C+ and D+) which were rewarded with 50% sucrose solution and two non-reinforced presentations of the compound stimulus (CD−). The stimuli were striped patterns (5 × 5 cm) subtending 64° to the bees’ eyes (Buatois et al. 2020). C+ and D+ consisted of either pure green or blue stripes respectively (RGB system: 0.255.0 and 0.0.255, see Fig. S4 for the spectral reflectance curves of the stimuli and their positions in the hexagon color space) on a black background while the compound CD- was composed of alternating blue and green stripes (Buatois et al. 2020). The colors of the stimuli were separated by 0.39 hexagon units, a color distance that granted color discrimination (Chittka 1992; Dyer and Neumeyer 2005; Avarguès-Weber et al. 2010). All stimuli had 5 colored stripes, whereas the outer two stripes were 0.6 cm wide and the three inner stripes were 1.2 cm wide. The stripes subtended a visual angle of 17° to the bees’ eyes, thus being perceived and discriminated based on their chromatic properties (Giurfa et al. 1996, 1997; Hempel de Ibarra et al. 2002). Each of the three stimulus types (C+ , D+ and CD−) had two variants with opposing stripe sequence to prevent learning based on fixed retinotopical images (Wehner 1972; Gould 1985; Giurfa et al. 1995). Stimuli were projected on a tracing paper screen with a video projector (Acer K1351, Acer Inc., Taiwan). The acquisition phase was followed by three consecutive non-reinforced tests, each presenting once each of the three stimuli.

### Statistical analysis

For individual analysis, a bee was characterized as ‘learner’, scored as 1 for the analysis, if it responded correctly in the test following each learning protocol (1st phase of reversal learning: response to A and not to B; 2nd phase of reversal learning: response to B and not to A; negative patterning: response to C and to D but not to CD, Mancini et al. 2018). All bees that exhibited other patterns of responses were considered as ‘non-learners’ and scored as 0 for the analysis. A more detailed analysis of the bees’ group acquisition and test performances can be found in the supplementary (Figs. S7 and S8). Unfortunately, we were not able to establish a satisfactory learning score from the acquisition phase to allow performances comparison between individuals. Indeed, scoring “1” each correct PER to the CS+ is not sufficient to characterize learning as both for the reversal learning paradigm and negative patterning paradigm, an absence of response to the CS- is also mandatory. Any arbitrary scoring method considering e.g. + 1 for a CS + response and −1 for a CS− response would lead to ambiguity in interpreting the resulting score. For example, a bee scored ‘0’ could have been none responsive to any stimulus or responsive to all stimuli. Individual consistency in learning performance was analyzed using Spearman rank correlations. Only bees characterized as ‘learners’ (*n* = 61 of 140 bees) in the test following the 1st phase of the reversal learning protocol were kept for analysis of the 2nd phase of reversal learning, as ‘success’ or ‘failure’ in the 2nd phase of reversal learning can only be assessed in bees that learned the initial association established in the 1st phase of reversal learning. Consequently, we could not correlate statistically the test performances of the 1st phase with those of the 2nd phase as correlations can only be performed when the data has more than one value (only learners of the first phase had to be used and in consequence all their responses were scored as 1). To assess if the order in which the experiments were conducted or the stimuli used (group_RL) had an influence on the test performances, GLMMs were used. The models with a binomial error structure and logit-link function included proboscis extensions made in the test (1 = PER, 0 = No PER) as dependent variable and the order of the tasks (*order*), the rewarded stimulus (*group_RL*) and the type of CS (*CS*) as fixed factors. The bees’ identity (*subject*) was included as a random factor. Different models were calculated by gradually removing factors and compared with an ANOVA. P-values from these comparisons were provided to account for each factor impact. The model with the lowest AIC value was chosen as most appropriate fit (see tables S16-18).

All GLMMs were performed using R Statistical Software version 3.6.3 (R Core Team 2022) with the package lme4 (Bates et al. 2015). All other statistical analyses and graphs were performed using GraphPad prism version 9.0.0 (GraphPad Software Inc., San Diego, California, USA). The significance level was *α* = 0.05 to account for our relatively small sample sizes (Lakens et al. 2018). For all correlations of the test performances across the three learning tasks, the null hypothesis was that the correlation coefficient rho was not different from zero.

### Results

**Fig. 4:**
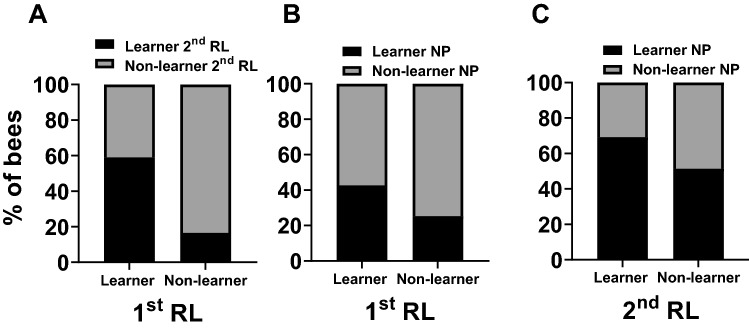
Correlations of the individual test performances in Experiment 3: Visual learning experiments with restrained bees. **A** A direct statistical correlation could not be performed, as we could only use the learner bees in the 1st phase as successful reversal of reward contingencies in the 2nd phase of reversal learning prerequisites learning the initial discrimination. We still observed that a majority of the learners in the 1st phase of reversal learning (1st RL) were also successful in the 2nd phase of reversal learning (2nd RL; 59%). 84% of the bees that failed to learn in the 1st phase, and were thus not used for the correlation, remained non-learners in the 2nd phase of reversal learning. **B** The individual test performances were positively correlated between the 1st phase of reversal learning and negative patterning (NP; Spearman rank correlation;* n* = 140, rho = 0.18, *p* = 0.03). Indeed, the majority of learners (57%) and non-learners (75%) in the 1st phase of reversal learning remained in their category in the negative patterning paradigm. **C** The individual test performances of the 2nd phase of reversal learning were not significantly correlated with negative patterning (Spearman rank correlation; *n* = 61, rho = 0.18, *p* = 0.17). While 69% of the learners in the 2nd phase of reversal learning were also successful in negative patterning, half of the bees (51%) that failed in the 2nd phase of reversal learning were nevertheless successful in negative patterning

We studied the learning performances of restrained bees (*n* = 140) conditioned with visual stimuli using a visual variant of the proboscis extension response (PER) protocol. Our goal was again to correlate performances across the two phases of reversal learning and between the reversal learning phases and negative patterning. Importantly, neither the order in which the learning tasks were trained (*order*), nor which stimulus was rewarded in reversal learning (*group_RL*) had a significant effect on the test results (GLMM: *order: n* = 140, **1**^**st**^** RL:** ${\chi }_{(1)}^{ 2}$ = 0.04, *p* = 0.8, **2**^**nd**^** RL:** ${\chi }_{(1)}^{ 2}$ = 0.002, *p* = 0.97, **NP:** ${\chi }_{(1)}^{ 2}$ = 0.04, *p* = 0.82, Tables S10–12; group_RL: *n* = 140, **1**^**st**^** RL:** ${\chi }_{(1)}^{ 2}$ = 0.84, *p* = 0.3, **2**^**nd**^** RL:** ${\chi }_{(1)}^{ 2}$ = 3.74, *p* = 0.06, **NP:** ${\chi }_{(1)}^{ 2}$ = 1.58, *p* = 0.21, Tables S16–S18). As mentioned above, success (‘learner’) or failure (‘non-learner’) in the 2nd phase of reversal learning is only informative if the bees successfully acquired the initial A+ B− discrimination in the 1st phase of reversal learning. Due to this, we could only use learner bees of the 1st phase (bees with score = 1) for any correlation involving the performances in the 2nd phase of reversal learning. As correlation analyses require at least two different values within each data frame we were mathematically unable to correlate performances of the 1st and the 2nd phase of reversal learning. Nevertheless, we observed that the majority of learners in the 1st phase was also successful in the 2nd reversal phase (59%, Fig. 4A). In the case of the excluded bees that were non-learners in the 1st phase, 84% (*n* = 66 out of 79 non-learners) in the 1st phase of reversal learning remained non-learners in the 2nd phase of reversal learning. Only 16% of the excluded non-learner bees (*n* = 13) learned to discriminate the two stimuli in the 2nd phase of reversal learning despite having failed to learn in the 1st phase. Individual test performances in the 1st phase of reversal learning and in negative patterning were significantly positively correlated (Spearman rank correlation; *n* = 140, rho = 0.18, *p* = 0.03, Fig. 4B, Table 1). No significant correlation was found between the test ranks of the 2nd phase of reversal learning and negative patterning (*n* = 61, rho = 0.18, *p* = 0.17, Fig. 4C, Table 1). This pattern of correlation could be intuited by the fact that only a minority of non-learner bees in the 1st phase of reversal learning was successful in the negative patterning protocol (25%), suggesting that being able to solve an elemental task might be a prerequisite to be able to solve negative patterning. By contrast, half of the bees (51%) that failed in 2nd phase despite being successful in the 1st phase of reversal learning were nevertheless successful in the negative patterning discrimination (Fig. 4B and C).

## Experiment 4. olfactory learning in restrained bees

### Material and methods

#### General method

Returning non-pollen foragers were caught at the entrance of a single hive in the morning of each experimental day. The bees were anaesthetized on crushed ice until they ceased their movements and were then harnessed individually in metal tubes so that only the mouthparts and antennae could be moved freely (Bitterman et al. 1983). The bees were fed with 2 µl of a sucrose solution (50%, w/w) and stored in a dark and humid box at room temperature for two hours before the start of the learning experiments. The experimental set-up for the two learning paradigms consisted of a bee holder facing olfactory stimulation and an air extractor providing a constant airflow behind the bees to avoid odors to stagnate. The odor stimulation was done manually using 20 ml syringes containing a filter paper soaked with 4 µl of the concerning odor. The timing of odor and sucrose stimulation as well as the inter-trial interval was controlled by the custom-written software program “TimingProtocol” (Lichtenstein et al. 2018).

Before the start of the learning experiments, the bees were tested for an intact PER by touching their antennae with a sucrose solution (50%, w/w). Only bees (*n* = 89) that responded with an extension of the proboscis were kept for the experiments. Additionally, the bees were tested for spontaneous PER to all conditioned odors. Bees that showed such spontaneous responses were not used for the experiments. Both learning protocols followed the same standardized methodology. Each conditioning trial lasted for 30 s and 10 bees were conditioned in “parallel”, i.e. they completed one trial, one after the other. In this way, the inter-trial-interval was five minutes. A trial started when a bee was positioned in front of the air extractor. After 14 s of familiarization with the experimental context, the odorant was delivered for four seconds. In rewarded trials, a toothpick soaked in sucrose solution was first delivered to the antennae to trigger the PER and then to the mouthparts so the bees could lick the solution for three seconds with one second overlap to odor stimulation. The toothpick was kept distant from bees before sucrose stimulation to avoid responses due to water vapor (Kuwabara 1957). During unrewarded trials, the timing remained identical, but no reward was given to the bees. After CS-US stimulation or CS stimulation only, the conditioned bee stayed in its position. Five minutes after completing each acquisition phase, two non-reinforced tests were conducted in which each CS was presented sequentially once without reinforcement. After the tests, the bees were checked again for intact PER. The order in which the stimuli were presented during the acquisitions and in the tests was pseudo-randomized. A conditioned response (a full extension of the proboscis beyond the imaginary line connecting the open mandibles) was recorded if the bee extended the proboscis (1 = response, 0 = no response) during the two seconds of odor stimulation. The two learning protocols were conducted on the same day spaced by one resting hour. Half of the bees were first subjected to the reversal learning protocol and then to negative patterning while the other half experienced the reversed sequence. Bees that did not respond to the sucrose solution with a proboscis extension during any acquisition trial were discarded from the analyses (*n* = *1*). Overall, the conditioning and testing phases lasted 5.5 h for 10 bees.

#### Reversal learning protocol

The bees were first subjected to the 1st phase of reversal learning during which one odorant was presented in association with a sucrose reward (A+) while a second odorant was not reinforced (B−). Two non-reinforced tests presenting sequentially stimuli A and B once, where conducted after the first acquisition phase. Thereafter, the 2nd phase was initiated after a resting time of 30 min. In this phase, the previously rewarded stimulus was unrewarded (A−) while the previously unrewarded stimulus was rewarded (B+). The reversal learning phase was again followed by non-reinforced tests presenting both stimuli A and B once. Both phases consisted of 5 CS+ trials and 5 CS− trials in a pseudo-random sequence. The two odorants were pure solutions of 2-hexanone and linalool (Sigma-Aldrich Chemie GmbH). The choice of A and B identity was balanced between odorants and bees.

#### Negative patterning protocol

During the acquisition phase, the bees were subjected to 20 trials divided into five blocks of four trials. One block consisted of one presentation of the odorant limonene (C+) paired with a sucrose reward, one presentation of the odorant 2-octanol (D+) also paired with sucrose and two presentations of the mixture (CD−), which was not reinforced. All odorants were obtained from Sigma-Aldrich Chemie GmbH. The sequence in which stimuli were presented was pseudo-randomized across the six blocks of trials. The subsequent non-reinforced retention tests consisted of one pseudo-randomized presentation of C, D and CD each, one after the other.

### Statistical analysis

For individual analysis, a bee was characterized as ‘learner’, scored as 1 for the analysis, if it responded correctly in the series of tests following each learning protocol (1st phase of reversal learning: response to A and not to B; 2nd phase of reversal learning: response to B and not to A; negative patterning: response to C and to D but not to CD, Mancini et al. 2018). All bees that exhibited other patterns of responses were considered as ‘non-learners’ and scored as 0 for the analysis. A more detailed analysis of the bees’ group acquisition and test performances can be found in the supplementary (Figs. S9 and S10). Unfortunately, we were not able to establish a satisfactory learning score from the acquisition phase to allow performances comparison between individuals. Indeed, scoring “1” each correct PER to the CS + is not sufficient to characterize learning as both for the reversal learning paradigm and negative patterning paradigm, an absence of response to the CS- is also mandatory. Any arbitrary scoring method considering e.g. + 1 for a CS + response and −1 for a CS− response would lead to ambiguity in interpreting the resulting score. For example, a bee scored ‘0’ could have been none responsive to any stimulus or responsive to all stimuli. Individual consistency in learning performance was analyzed using Spearman rank correlations. Only bees characterized as ‘learners’ (*n* = 42 of 89 bees) in the test following the 1st phase of the reversal learning protocol were kept for analysis of the 2nd phase of reversal learning, as ‘success’ or ‘failure’ in the 2nd phase of reversal learning can only be assessed in bees that learned the initial association established in the 1st phase of reversal learning. Consequently, we could not correlate statistically the test performances of the 1st phase with those of the 2nd phase as correlations can only be performed when the data has more than one value (only learners of the first phase had to be used and in consequence all their responses were scored as 1). To assess if the order in which the experiments were conducted or the stimuli used (group_RL) had an influence on the test performances, GLMMs were used. (see the statistical analysis paragraph of experiment 3 for a detailed description of the GLMMs and model selection procedure, Tables S22–S24).

### Results

We subjected restrained bees (*n* = 89) to olfactory PER conditioning and determined if performances in the two phases of a reversal learning problem were correlated with performances in a negative-patterning problem. Again, the sequence in which the problems were trained (*order*) and the stimulus identity in reversal learning (*group_RL*) had no influence on the test performances (GLMM: Order: *n* = 89, **1**^**st**^** RL:** ${\chi }_{(1)}^{ 2}$ = 3.07, *p* = 0.08, **2**^**nd**^** RL:** ${\chi }_{(1)}^{ 2}$ = 0.10, *p* = 0.75; **NP:** ${\chi }_{(1)}^{ 2}$ = 0.51, *p* = 0.47, Tables S22–S24; group_RL: *n* = 89, **1**^**st**^** RL:** ${\chi }_{(1)}^{ 2}$ = 0.01, *p* = 0.9, **2**^**nd**^** RL:** ${\chi }_{(1)}^{ 2}$ = 0.76 *p* = 0.38, **NP:** ${\chi }_{(1)}^{ 2}$ = 0.13, *p* = 0.72, Tables S22–S24).

**Fig. 5:**
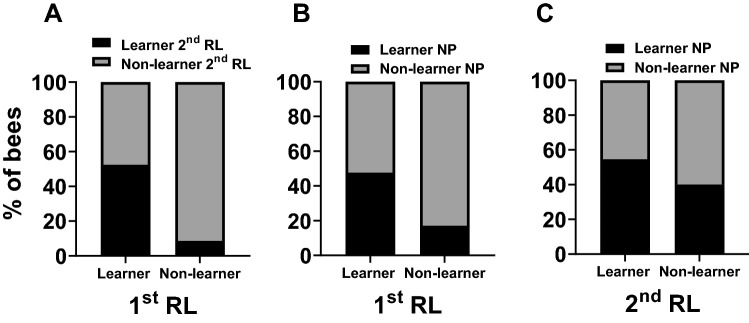
Correlations of the individual test performances in Experiment 4: Olfactory learning experiments with restrained bees. **A** A direct statistical correlation could not be performed, as we could only use the learner bees in the 1st phase as successful reversal of reward contingencies in the 2nd phase of reversal learning prerequisites learning the initial discrimination. We still observed that many of the learners in the 1st phase of reversal learning (1st RL) were also successful in the 2nd phase of reversal learning (2nd RL; 53%). 91. % of the bees that failed to learn in the 1st phase, and were thus not used for the correlation, were also non-learners in the 2nd phase of reversal learning. **B** The individual test performances were positively correlated between the 1st phase of reversal learning and negative patterning (2nd RL; Spearman rank correlation; *n* = 89, rho = 0.33, *p* = 0.002). Indeed, many learners (48%) and non-learners (83%) in the 1st phase of reversal learning remained in their category in the negative patterning paradigm. **C** The individual test performances of the 2nd phase of reversal learning were not significantly correlated with negative patterning (NP; Spearman rank correlation; *n* = 42, rho = 0.15, *p* = 0.36). While 55% of the learners in the 2nd phase of reversal learning were also successful in the negative patterning task, almost half of the bees (40%) that failed in the 2nd phase of reversal learning were nevertheless successful in the negative patterning task

To analyze individual consistency across tasks including the 2nd phase of reversal learning, and for the reasons already explained, we used only bees that learned the discrimination of the 1st phase of reversal learning (*n* = 41). As in the experiment on visual PER conditioning, we could not correlate statistically the individual test performances of the 1st and 2nd phase of reversal learning in these bees. Yet, half of learners in the 1st phase (52%) were also successful in the 2nd phase of reversal learning (Fig. 5A). In the case of the bees that were excluded as non-learners due to their performance in the 1st phase, 91% (*n* = 43 out of 47) remained non-learners in the 2nd phase of reversal learning. Test performances were significantly positively correlated in the 1st phase of reversal learning and in negative patterning (Spearman rank correlation; *n* = 89, rho = 0.33, *p* = 0.002, Fig. 5B, Table 1). However, there was no significant correlation between test performances in the 2nd phase of reversal learning and negative patterning (*n* = 42, rho = 0.15, *p* = 0.36, Fig. 5C, Table 1). As in the experiment on visual PER conditioning, this lack of correlation is mainly due to the fact that while only 17% of non-learners in the 1st phase of reversal learning learned successfully the negative patterning problem, 40% of the non-learner bees in the 2nd phase of reversal learning succeeded in negative patterning (Fig. 5B and C).

## Discussion

We focused on individual learning performances of honey bees to determine if learning proficiency is maintained at the individual level across learning tasks differing in cognitive complexity and processing (elemental or non-elemental discriminations requesting cognitive flexibility or configural abilities). We replicated this analysis using discrimination tasks involving different sensory modalities (olfaction or vision), distinct types of conditioning (Pavlovian, in the case of harnessed bees, or a combination of operant and Pavlovian in the case of free-flying bees) and various experimental set-ups and restricting or not freedom of movement (rotating screen or Y-maze for free-flying conditions; restrained conditions in PER conditioning experiments or free movement in free-flying experiments). Across these multiple scenarios, learning performances exhibit appreciable inter-individual variation. Interestingly, the individual performances remained consistent across some, but not all protocols tested. The individual bees’ proficiency to solve an elemental association in the 1st phase of reversal learning (A+ vs. B−) correlated positively with the performance in the 2nd phase (A− vs. B+ , Table 1) and negative patterning (C+ and D+ vs. CD−, Table 1). However, we did not find a significant correlation between performances in the 2nd phase of reversal learning and negative patterning, i.e. between the task that requires overcoming transient stimulus ambiguity and the configural task, respectively (Table 1). Interestingly, this pattern of correlation was stable irrespective of the training method and the sensory modality. We therefore conclude that the pattern of correlations observed is a real characteristic of the bees’ cognitive profile.

Studies on bumble bees (*Bombus terrestris*) and fruit flies (*Drosophila melanogaster*) have also demonstrated a positive association between the 1st and 2nd phase of a reversal learning problem within a single modality (bumble bees, visual learning: Raine and Chittka 2012; fruit flies, olfactory learning: Smith et al. 2022). We now extend this conclusion to honey bees tested in two sensory modalities with either Pavlovian or operant-Pavlovian conditioning (i.e. using harnessed bees or free-flying bees, respectively). In a previous study (Finke et al. 2021), we also showed that the bees’ performance in an elemental visual discrimination task was positively correlated with the performance in a higher-order visual task, a relational concept learning task. In the present study we found no significant association between the individuals’ performances in the 2nd phase of reversal learning and negative patterning, suggesting that bees might specialize in some cognitive trait either independently or at the expense of other faculties. Indeed, a trade-off in the performances of bees has been found between appetitive and aversive learning (Junca et al. 2019) or between olfactory and landmark learning (Tait et al. 2019). Moreover, no correlation, be it positive or negative, was found between visual and olfactory elemental learning (honey bees: Finke et al. 2021; bumble bees: Smith and Raine 2014).

Although cognitive specialization may account for the lack of significant correlation between reversal learning and negative patterning, an alternative hypothesis could be that different strategies are used by individual bees to solve non-elemental problems without being necessarily the consequence of an absence of competence (Komischke et al. 2003; Dyer et al. 2014). Additionally, modifications of motivational state and attention between the different experimental phases may lead to higher variability in the performances which may conceal individual consistency across these tasks. However, this last explanation should only act marginally as we found strong stability of performances across time within a given type of learning task in bees (Finke et al. 2021).

A long-standing question in cognitive sciences is whether cognition is composed of specialized modules that evolved independently of each other, or if and to what extent a general factor (termed ‘factor g’ by Charles Spearman) accounts for consistent inter-individual variability across multiple cognitive performances (Spearman 1904). The theory of general intelligence, which has been extensively studied in humans and other vertebrates, considers that the performances in multiple cognitive tests are highly correlated given that the g-factor accounts for a large proportion of inter-individual variability in these tests (Jensen 1998; Mackintosh 1998; Plomin and Spinath 2002; Matzel et al. 2003; Galsworthy et al. 2005; Brown and Price 2007; Herrmann and Call 2012). In humans, the g-factor has been correlated with brain parameters and function such as brain size, gray matter substance, cortical thickness, or processing efficiency (Jung and Haier 2007; Deary et al. 2010). These results suggest that mechanisms of general information processing represent an important part of multiple correlated cognitive performances (Deary et al. 2010). In recent years, the topic of domain-general cognition has gained increasing interest in the field of insect neurobiology although studies comparing the performances of individual insects across tasks with distinct cognitive demands are still missing (Simons and Tibbetts 2019). Besides the correlation between the performances in the 1st and 2nd phase of reversal learning found in bees and flies (see above), positive correlations across tasks have also been found between latent inhibition and reversal learning (Chandra et al. 2000) or between an elemental discrimination and a non-elemental concept learning task (Finke et al. 2021) and here between an elemental discrimination and reversal learning or configural learning. Additionally, in some insect species the learning performance was consistent over time (honey bees: Finke et al. 2021; fruit flies: Smith et al. 2022) and across visual, olfactory and tactile elemental discriminations (bumble bees: Muller and Chittka 2012). Although our data do not provide enough evidence for significant positive correlations between the 2nd phase of reversal learning and negative patterning, it might still be possible that a g-factor accounts for a small proportion of inter-individual variability across the three learning tasks tested but being concealed by inter-individual variability caused by other experimental or intrinsic factors and low sample sizes. Inter-individual differences in cognitive performances have been related in some cases with inter-individual differences in neural anatomy and processing in insect brains (Li et al. 2017; Honegger et al. 2019; Linneweber et al. 2020) providing clear demonstrations of how individuality in behavior can be directly traced back to individual differences in neuronal processing in insects. An alternative explanation to the consistent performance observed within individuals despite the variability existing between individuals might refer to differences in reinforcement motivation. For example, in bees, sucrose responsiveness, which is used to measure individual sensitivity to the sucrose reward, correlates positively with individual performances in elemental appetitive learning tasks, i.e. bees that show a higher responsiveness and thus responding to a broad spectrum of sucrose concentrations generally learn better than bees with lower responsiveness; Scheiner et al. 1999, 2001a, b, 2005). Since we used sucrose as reward in all our learning tasks, we cannot exclude that a proportion of the inter-individual variability in learning performances observed in our experiments could be attributed to differences in sucrose responsiveness. Yet, this factor cannot fully account for the consistent differences in individual learning performances reported here. Indeed, if it had played a major role, we would have found universal positive correlations across all tasks tested, which was not the case.

Contrary to the theory of general intelligence, the theory of domain-specific cognition postulates that cognition is modular, meaning that distinct mental or cognitive modules rely on specialized mechanisms used to solve specific problems evolving independently (Friederici 1990; Sperber 1994; Shettleworth 2000; Palmer and Palmer 2002). This hypothesis seems confirmed in honey bees by the existence of a trade-off between appetitive and aversive learning capabilities (Junca et al. 2019) despite previous negative results (Roussel et al. 2009) or between olfactory elemental learning and landmark learning (Tait et al. 2019) and a lack of correlation between visual and olfactory learning (Finke et al. 2021) or here between reversal learning and negative patterning.

Finally, a third theory postulates the co-existence of domain-general and independent domain-specific cognitive modules (Plomin 2001; Brown and Price 2007). Indeed, in humans, some general properties of the brain (e.g. amount of grey matter, processing speed) have general effects on different brain regions and thereby lead to positive correlations among performances in different cognitive domains, even though their specific mechanisms are located in distinct regions of the brain (Jensen 1993; MacLullich et al. 2002; Lee 2007). In honey bees, the adoption of this view was proposed by Menzel and Giurfa (2001), who referred these different levels of modularity to specific brain areas based on their cross-modality or insulation from other processing pathways. For instance, while some olfactory learning forms can be mediated by neural phenomena in pure olfactory regions and circuits (Faber et al. 1999; Rath et al. 2011), being therefore domain-specific, other learning forms require multi-modal regions such as the mushroom bodies, which can be seen as domain-general modules. Thus, domain-specific and domain-general mechanisms may interact to mediate the cognitive abilities in insects. We could thus hypothesize that general stimulus processing abilities within a given sensory modality, general brain structure and metabolism or attentional and working memory capacities mediate cognition in a domain-general manner, thereby deeply influencing performances in any learning task with a given sensory modality or reinforcement type such as found in humans (Chiappe and MacDonald 2005; Matzel and Kolata 2010; Kanai and Rees 2011; Völter et al. 2018). Different regions in the bee brain have been associated with different forms of learning based on the level of stimuli ambiguity: while the mushroom bodies are dispensable for solving elemental discriminations (Malun et al. 2002; Komischke et al. 2005; Devaud et al. 2007), they are indispensable for solving learning tasks with transient or permanent stimuli ambiguity (i.e. reversal learning: Devaud et al. 2007; Boitard et al. 2015; Negative patterning: Devaud et al. 2015). In parallel, the seemingly lack of correlation between reversal learning and negative performance may be due to individual differences in neuronal circuits specialized in these different tasks. While the mushroom bodies are necessary for acquiring non-elemental olfactory tasks both in a negative patterning paradigm (Devaud et al. 2015) and a reversal learning problem (Boitard et al. 2015), the specific neurons involved may be different.

An intriguing perspective of our findings is why stable individual differences in learning proficiency would be maintained within colonies. One potential explanation relies on the potential costs of cognitive functions (Mery and Kawecki 2003, 2004; Mery 2005): One could hypothesize that increased ability for a cognitive trait might trade-off against the ability for another trait (Chittka et al. 2003; Hollis and Guillette 2015; Tait et al. 2019; Junca et al. 2019). Consequently, different cognitive abilities in different individuals could lead to task specialization or specific adaptation to given environmental conditions. However, it remains understudied whether and to what extent inter-individual cognitive variability contributes to fitness and survival of animals (Thornton et al. 2014; Cauchoix and Chaine 2016; Cauchoix et al. 2018). It is often assumed that a mixture of individual strategies in behavioral traits could influence the flexibility of colonies to react to changing environmental conditions (Burns and Dyer 2008; Dyer et al. 2014; Jandt et al. 2014). However, evidence supporting that inter-individual variability in learning performance among workers accounts for differences in their foraging success or their foraging behavior (e.g. scouts and recruits; Beekman et al. 2007) are still lacking. In bumble bees, colony variation in learning speed in an elemental visual discrimination task was correlated with their foraging success under natural conditions (Raine and Chittka 2008). However, Evans et al. (2017) found that fast and slow bumble bee learners had comparable rates of food collection and even that bees with higher learning proficiency foraged for shorter periods compared to those with lower learning abilities. These results might be the consequence of higher metabolic costs of increased learning proficiency. In any case, more research is thus necessary to link the cognitive abilities of individuals and their level of variability within a hive to their foraging performance (i.e. amount of food resources collected) in the field and under different scenarios of resources availability and distribution.

## Supplementary Information

Below is the link to the electronic supplementary material.

Supplementary file1 (DOCX 897 KB)

## Data Availability

The raw data are available on figshare: https://doi.org/10.6084/m9.figshare.20473113.v.
