# Supplementary material for: Individual consistency in the learning abilities of honey bees: cognitive specialization within sensory and reinforcement modalities
Source: Anim Cogn. 2023 Jan 6;26(3):909–28. doi: 10.1007/s10071-022-01741-2 (PMC10066154; doi:10.1007/s10071-022-01741-2)
Supplement: Supplementary file 1 — Supplementary file1 (DOCX 897 KB) [file 10071_2022_1741_MOESM1_ESM.docx]

**Supplementary material**

**Individual consistency in the learning abilities of honey bees: Cognitive specialization within sensory and reinforcement modalities**

Valerie Finke^1,2^, Ricarda Scheiner^1^, Martin Giurfa^2,3^ and Aurore Avarguès-Weber^2^

*^1^Biozentrum, Universität of Würzburg, Am Hubland, 97074 Würzburg, Germany*

*^2^Centre de Recherches sur la Cognition Animale (CRCA), Centre de Biologie Intégrative (CBI), Université de Toulouse; CNRS, UPS, 118 Route de Narbonne, 31062 Toulouse, France*

*^3^Institut Universitaire de France, Paris, France*

**Corresponding author :** Valerie Finke – [valerie.finke@uni-wuerzburg.de](mailto:valerie.finke@uni-wuerzburg.de)

**Analysis of group performances**

**Statistical analysis**

All acquisition performances were analyzed using generalized linear mixed models (GLMM) for repeated measurements with a binomial family including trial number, trial identity (reinforced/non-reinforced/punished for experiments 3 and 4 only), rewarded stimulus (group_RL) and which learning task was conducted first (negative patterning or reversal learning first; order) as fixed factors, subject as a random factor and the individual bee responses (correct = 1 or incorrect = 0) as dependent variable. We also included an interaction between trial identity and trial number. Different models were performed where the factors were gradually removed and compared using an ANOVA. The model with the lowest AIC value was chosen as most appropriate fit (Burnham and Anderson 1998; Panchal et al. 2010; see tables S1-S18). GLMMs were performed using R Statistical Software (v3.6.3; R Core Team 2022) with the package lme4 (Bates et al. 2015).

**Experiments 1 & 2: Operant conditioning experiments with free-flying bees**

Group acquisition curves of the learning tasks with free-flying bees were created by calculating the percentage of correct choices of all tested bees for each trial. For negative patterning, the acquisition curves consisted solely of the trials with the mixture since the trials with the single stimuli were conducted with absolute conditioning. A paired t-test was used to assess if the bees had learnt the task in each test. Test performances were also analyzed using GLMM with a binomial error structure and logit-link function. The responses (1 = correct, 0 = incorrect) of the bees in the 20 non-reinforced trials in the tests following each learning task were the dependent variable while rewarded stimulus (group_RL) and which learning task was conducted first (negative patterning or reversal learning first; order) as fixed factors. Subject identity was included as a random factor.

**Experiments 3 & 4: Classical conditioning with restrained bees**

The group acquisition curves of the learning protocols with restrained bees were generated by calculating the proportion of bees that showed the conditioned response upon stimulus presentation during each trial (reversal learning) or block of trials (negative patterning). For the test performances we calculated the percentage of bees showing PER upon stimulus presentation. To evaluate if the bees had learnt the task, test performances were analyzed using a McNemar test. Test performances were also analyzed using GLMM with a binomial error structure and logit-link function. The responses (PER = 1, no PER = 0) of each bee to the stimuli in the non-reinforced tests of each learning task were the dependent variable while rewarded stimulus (group_RL), which learning task was conducted first (negative patterning or reversal learning first; order) and the identity of the CS (CS) were included as fixed factors. Subject identity was included as a random factor.

The bees were also grouped according to their test performance: A bees’ performance was characterized as ‘correct response’ if it responded fully correct in the test (1^st^ phase of reversal learning: Response to A and not to B, 2^nd^ phase of reversal learning: Response to B and not to A, Negative patterning: Response to C and D not to CD), as ‘incorrect response’ if it responded to all stimuli or only to the unrewarded stimulus and as ‘no response’ if it did not respond to any stimulus but showed an intact PER to sucrose after the test. Then, we compared the test groups of the bees in the different tests, by calculating the relative percentage of bees belonging to the three test categories in a given learning task in relation to their performance in an alternative task (Figures S6 for experiment 3 and S8 for experiment 4).

**Results**

**Experiment 1: Visual learning experiments with free-flying bees**

The bees’ group acquisition performances improved significantly across the 1^st^ phase of reversal learning (1^st^ RL), the 2^nd^ phase of reversal learning (2^nd^ RL) and negative patterning (GLMM, *Trial*; N = 33; **1^st^ RL**: χ^2^ = 27.24, df = 1, p < 0.0001, Fig. S3A, Table S1; **2^nd^ RL**: χ^2^ = 27.1, df = 1, p < 0.0001, Fig. S3B, Table S2; **NP**: χ^2^ = 64.60, df = 1, p < 0.0001, Fig. S3C, Table S3). Similarly, the bees’ learnt to choose the rewarded stimuli significantly more often than the punished stimuli in both phases of reversal learning (Paired t-test, N = 33, **1^st^ RL:** 71.36 ± 2.43 % of correct choices; t = 8.81, df = 32, p < 0.0001, Fig. S3A; **2^nd^ RL**: 57.88 ± 2.4 % of correct choices; t = 3.28, df = 32, p = 0.003, Fig. S3B). As the performances of the bees in the two retention tests of the negative patterning protocol were not significantly different, they were pooled for analysis (Paired t-test; N = 33, 69.85 ± 2.05 % choices for the blue checkerboard C+ over CD- and 66.52 ± 1.98 % for the pink checkerboard D+ over CD; t = 1.89, df = 32, p = 0.07). The bees learnt to choose the single stimuli significantly more often than their mixture (Paired t-test, N = 33; 68.18 ± 1.81 % choices for C+/D+; t = 9.97, df = 32, p < 0.0001, Fig. S3C). The color used as target stimulus (68N or 3N) in reversal learning did not affect the acquisition performances nor the test performances (Acquisition: GLMM, *group_RL, N = 33,* ***1^st^ RL****:* χ^2^ = 0.01, df = 1, p = 0.91, Table S1; ***2^nd^ RL****:* χ^2^ = 0.04, df = 1, p = 0.85, Table S2; ***NP****:* χ^2^ = 0.01, df = 1, p = 0.92, Table S3; Test: GLMM, *group_RL, N = 33,* ***1^st^ RL****:* χ^2^ = 2.1, df = 1, p = 0.08, Table S4; ***2^nd^ RL****:* χ^2^ = 1.41, df = 1, p = 0.24, Table S5; ***NP****:* χ^2^ = 0.15, df = 1, p = 0.70, Table S6). The order in which the learning protocols were conducted had only a significant effect on the acquisition performances of the 2^nd^ phase of reversal learning (GLMM; *order*, N = 33, ***1^st^ RL****:* χ^2^ = 3.29, df = 1, p = 0.07, Table S1; ***2^nd^ RL****:* χ^2^ = 6.27, df = 1, p = 0.01, Table S2; ***NP****:* χ^2^ = 0.08, df = 1, p = 0.78, Table S3).

**Experiment 2: Olfactory learning experiments with free-flying bees**

Throughout the acquisition phases of the two learning protocols the bees’ group performance increased significantly (GLMM; *trial,* N = 22; **1^st^ RL**: χ^2^ = 38.71, df = 1, p < 0.0001, Fig. S4A; Table S7; **2^nd^ RL**: χ^2^ = 45.11, df = 1, p < 0.0001, Fig. S4B, Table S8; **NP**: χ^2^ = 28.92, df = 1, p < 0.0001, Fig. S4C, Table S9). In the retention tests of the two phases of the reversal learning paradigm the bees chose the correct stimulus significantly more often than the incorrect option (Paired t-test, N = 22; **1^st^ RL**: 81.95 ± 2.96 % of correct choices; t = 10.79, df = 21, p < 0.0001, Fig. S4A; **2^nd^ RL**: 73.45 ± 3.99 % of correct choices; t = 5.68, df = 21, p < 0.0001, Fig. S2B). The performance of the bees in the two retention tests of negative patterning did not differ and were thus pooled for analysis (Wilcoxon signed rank test, N = 22; 76.05 ± 3.27 % choices for Limonene vs. the mixture and 75.09 ± 5.3 % choices for 2-Octanol vs. the mixture; W = 8, p = 0.86). The bees significantly preferred the single stimuli over their mixture in the non-reinforced tests (Paired t-test, N = 22; 75.57 ± 3.84 % choices for C+/D+; t = 6.46, df = 21, p < 0.0001, Fig. S4C). The rewarded stimulus used in reversal learning did not affect the bees’ acquisition performances (Acquisition: GLMM, N = 22, *Stimuli;* **1^st^ RL**: χ^2^ = 0.63, df = 1, p = 0.43, Table S7; **2^nd^RL**: χ^2^ = 0.37, df = 1, p = 0.53, Table S8; **NP**: χ^2^ = 2.22, df = 1, p = 0.14, Table S9; Test: GLMM, N = 22, **1^st^ RL**: χ^2^ = 0.17, df = 1, p = 0.68, Table S10; **2^nd^ RL**: χ^2^ = 0.35, df = 1, p = 0.55, Table S11; **NP**: χ^2^ = 1, df = 1, p = 0.32).

**Experiment 3: Visual learning experiments with restrained bees**

The bees’ group performance increased significantly throughout the acquisition of all tasks (GLMM: *Trial*; N = 140; **1^st^ RL**: χ^2^ = 66.04, df = 1, p < 0.0001, Fig. S5A Table S13; **2^nd^ RL**: χ^2^ = 164.5, df = 1, p < 0.0001, Fig. S5B, Table S14; **NP**: χ^2^ = 168.3, df = 1, p < 0.0001, Fig S5B, Table S15). In the non-reinforced tests following each phase of the reversal learning protocol the bees’ showed the ability to discriminate the rewarded from the non-rewarded stimulus (McNemar test, N = 140; **1^st^ RL**: 62.1 % of the bees showing a PER to A+ and 21.4 % to B-; χ^2^ = 48.25, df = 1, p < 0.0001, Fig S5A; **2^nd^ RL**: 60.7 % of the bees showing a PER to B+ and 38.6 % to A-; χ^2^ = 13.43, df = 1, p < 0.0001, Fig. S5B). As bees did not respond differently to C and D in the test of the negative patterning protocol, they were pooled for analysis (McNemar test; N = 140; 66.4 % of the bees responding to C and 67.9 % responding to D; χ^2^ = 0.06, df = 1, p = 0.81). Taking the pooled data into account the bees significantly preferred the single stimuli C/D over the compound CD in the acquisition and in the non-reinforced test of the negative patterning paradigm (Acquisition: GLMM, N = 140; *CS*: χ^2^ = 188.5, df = 1, p < 0.0001, Fig. S5C, Table S15; Retention test: McNemar test; N = 140, χ^2^ = 10.4, df = 1 p < 0.001, Fig. S5C). The order in which the learning paradigms were conducted had a significant effect on the acquisition performances of the 2nd phase of reversal learning only (Acquisition: GLMM, N = 140; *Order*: **1^st^ RL:** χ^2^ = 0.12, df = 1, p = 0.73, Table S13; **2^nd^ RL:** χ^2^ = 164.5, df = 1, p < 0.0001, Table S14; **NP:** χ^2^ = 3.38, df = 1, p = 0.07, Table S15). There was a significant interaction effect between the trials, the stimuli used and CS across all acquisition phases (Acquisition: GLMM, N = 140, *trials*group_RL*CS*: **1^st^ RL:** χ^2^ = 40.53, df = 1, p < 0.0001, Table S13; **2^nd^ RL:** χ^2^ = 164.5, df = 1, p < 0.0001, Table S14; **NP:** χ^2^ = 188.5, df = 1, p < 0.0001, Table S15).

**Experiment 4: Olfactory learning experiments with restrained bees**

The bees’ group performance increased significantly throughout the acquisition phases of all tasks (GLMM: N = 89; *Trial*; **1^st^ RL**: χ^2^ = 274.1, df = 1, p < 0.0001, Fig. S7A; **2^nd^ RL**: χ^2^ = 291.8, df = 1, p < 0.0001, Fig. S7B; **NP**: χ^2^ = 293.9, df = 1, p < 0.0001, Fig. S7C; Tables S19, S20 and S21). Similarly, the bees showed the ability to discriminate the two stimuli in the tests of the reversal learning protocol (McNemar test; N = 89; **1^st^ RL**: 62.92 % of bees responding to A+ and 17.98 % responding to B-; χ^2^ = 34.57, df = 1, p < 0.0001, Fig. S7A; **2^nd^ RL**: 61.8 % responding to B+ and 36 % to B-; χ^2^ = 16.69, df = 1, p < 0.0001, Fig. S7B). In the test of negative patterning the bees did not respond differently to the two single stimuli C and D and therefore the results were pooled for analysis (McNemar test, N = 89; 65.17 % of the bees responding to Limonene and 56.18 % responding to 2-Octanol; χ^2^ = 3.36, df = 1, p = 0.07). The pooled data shows that the bees significantly preferred the correct single stimuli C and D compared over the incorrect mixture CD in the test (60.67 % of bees responding to C+/D+ and 20.23 % responding to CD-; McNemar test; χ^2^ = 29.26, df = 1, p < 0.0001, Fig. S7C). The order in which the learning paradigms were conducted had a significant effect on the acquisition performances of the 1st phase of reversal learning only (Acquisition: GLMM, N = 89; *Order*: **1^st^ RL:** χ^2^ = 5.34, df = 1, p = 0.03, Table S19; **2^nd^ RL:** χ^2^ = 3.83, df = 1, p = 0.06, Table S20; **NP:** χ^2^ = 1.6, df = 1, p = 0.21, Table S21). There was a significant interaction effect between the trials, the stimuli used and CS across all acquisition phases (Acquisition: GLMM, N = 89, *trials*group_RL*CS*: **1^st^ RL:** χ^2^ = 232.1, df = 1, p < 0.0001, Table S19; **2^nd^ RL:** χ^2^ = 239.9, df = 1, p < 0.0001, Table S20; **NP:** χ^2^ = 239, df = 1, p < 0.0001, Table S21).

**Supplementary figures**

**
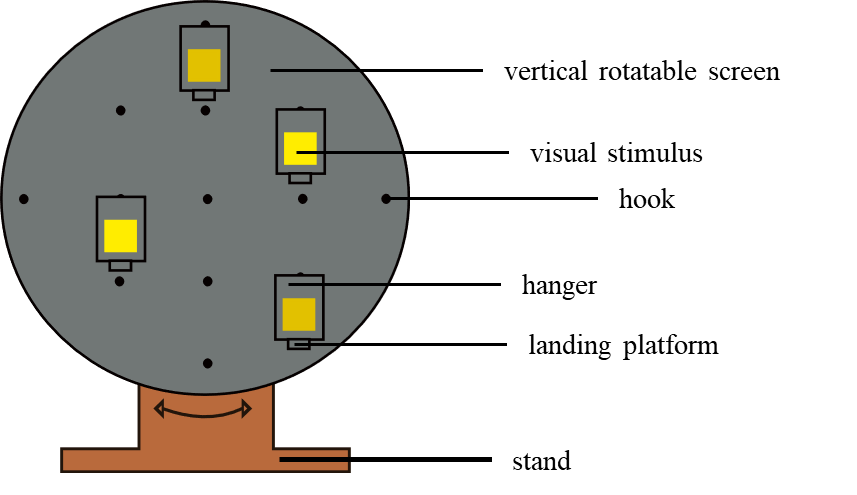
**

***Fig. S1*** *Schematic overview of the rotating screen apparatus used in experiment 1: Visual learning experiments with free-flying bees. The rotating screen consisted vertical rotatable screen (50 cm in diameter) connected to a stand. The screen displayed hooks at various locations where hangers (6 x 8 cm) could be attached to. During conditioning and testing of the two learning tasks the hangers displayed the visual stimuli (5 x 5 cm). During conditioning the reinforcements of the stimuli could be collected by the bees from the landing platform of the respective hangers. Each stimulus was presented twice on the rotating screen.*

***Fig. S2*** *Spectral properties of the colored stimuli used in reversal learning and negative patterning of experiment 1. The spectral curves were measured with a spectrophotometer (Avantes AvaSpec-ULS2048L). The reflectance curves for the HKS paper stimuli were measured with the Avalight Xenon source and FCR-7UV200-2-M2 optical fiber. The hexagon color space is a model representing color representation from photoreceptor excitation based on generalized color opponency. Perceptual discrimination between colors could be quantify via the Euclidean distance between colored stimuli (Chittka 1992). For the calculations of the hexagon distances of the stimuli, the spectral sensitivities of the honey bee photoreceptors (Peitsch et al. 1992), a standard daylight function D65 (Judd et al. 1964) and the grey background of the hangers were used.* ***A)*** *Spectral reflectance curves of the yellow (3N) and greenish-yellow (68N) stimuli used in reversal learning and of the pink (26N), blue (44N), black (88N) and grey (92) colors used to create the stimuli in negative patterning****. B)*** *Loci of the colored stimuli in a hexagon color space for the trichromatic color vision of honey bees. The distances of the stimuli used in reversal learning (3N and 68N) were 0.07 hexagon units and 0.07 for the colors used in negative patterning (26N and 44N). The mean distance between all stimuli used in experiment 1 was 0.30 ± 0.04 hexagon units.*

*
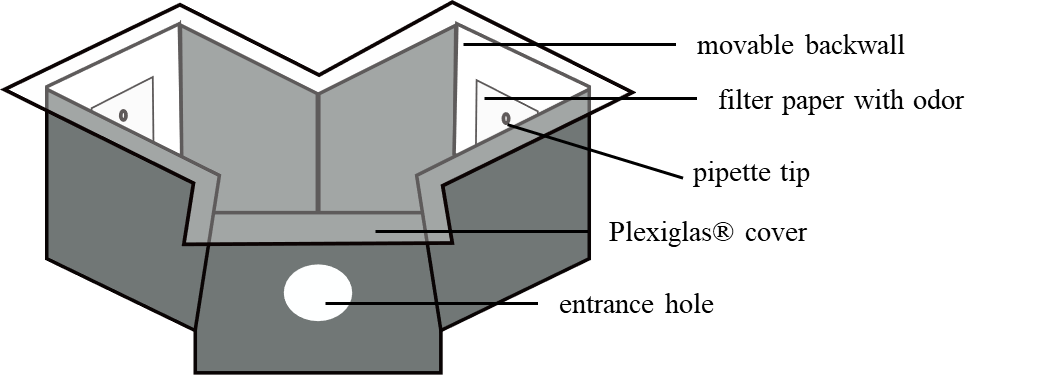
*

***Fig. S3*** *Schematic overview of the Y-maze apparatus used in experiment 2: Olfactory stimuli and free-flying bees. The maze could be entered by free-flying bees through an entrance hole leading to the two arms of the maze (40 x 20 x 20 cm). The backwalls (20 x 20 cm), coated with white copy paper, displayed the stimuli during conditioning and testing. The stimuli in this experiment were different odors applied to a filter paper (5 x 5 cm) and attached to the backwalls. The reinforcement was provided inside micro pipette tips located in the middle of each backwall. The Y-maze was covered by a UV-transparent Plexiglas® ceiling.*

***Fig. S4*** *Spectral properties of the colored stimuli used in reversal learning and negative patterning of experiment 3. The spectral curves were measured with a spectrophotometer (Avantes AvaSpec-ULS2048L). The irradiance curves for the coloured light stimuli were measured with the FC-UV400-2-SR optical fiber after calibration of the spectrophotometer with an halogen source (AvaLight-Hal-Cal-MINI), Ocean Optics SD2000 with a DT1000 mini light source (200–1,100 nm) and R400-7 UV/VIS optical fibre. The hexagon color space is a model representing color representation from photoreceptor excitation based on generalized color opponency. Perceptual discrimination between colors could be quantify via the Euclidean distance between colored stimuli (Chittka 1992). For the calculations of the hexagon distances of the stimuli, the spectral sensitivities of the honey bee photoreceptors (Peitsch et al. 1992) and the residual black light of the room were used.* ***A)*** *Normalized irradiance curves of the monochromatic stimuli (generated by a polychromator) used in reversal learning (400nm and 600 nm) and of the blue and green colors (generated by a video projector) used to create the stimuli in negative patterning.* ***B)*** *Loci of the colored stimuli in a hexagon color space for the trichromatic color vision of honey bees. The distances of the stimuli used in reversal learning (400 and 600 nm) were 0.67 hexagon units and 0.39 for the colors used in negative patterning (blue and green). The mean distance between all stimuli used in experiment 3 was 0.42 ± 0.01 hexagon units.*

***Fig. S5*** *Group acquisition curves and group test performances of the reversal learning (A+B) and negative patterning (C) protocols in experiment 1 (N =33).* ***A)*** *1^st^ phase of the reversal learning protocol. The curve shows the mean percentage of correct choices of the group for the rewarded stimulus (A+) during the 30 trials of the acquisition phase. The test performance shows the mean percentage of choices for the rewarded stimulus (A+; black bar) and the punished stimulus (B-; grey bar) during the 20 non-reinforced choices of the test,* ***B)*** *2^nd^ phase of the reversal learning protocol. The curve shows the mean percentage of correct choices of the group for the rewarded stimulus (B+) during the 30 trials of the acquisition. The histogram shows the mean percentage of choices for the correct (B+; black bar) and incorrect stimulus (A-; grey bar) during the 20 non-reinforced choices of the test and* ***C)*** *Negative patterning protocol. The curve shows the mean percentage of choices of the group for the incorrect mixture stimulus (CD-) throughout the 60 trials the acquisition phase. As the test performances of the two single stimuli (C+ and D+) did not differ from each other, they were pooled for analysis. The histogram shows the mean percentage of choices pooled for the correct two single stimuli (C+/D+; black bar) and the incorrect mixture stimulus (CD-; grey bar) during the 20 choices of the unreinforced tests. **p ≤ 0.01, ***p ≤ 0.001*

***Fig. S6*** *Group acquisition curves and group test performances of the reversal learning (A+B) and negative patterning (C) protocols in experiment 2 (N=22).* ***A)*** *1^st^ phase of the reversal learning protocol. The curve shows the mean percentage of correct choices of the group for the rewarded stimulus (A+) during the 10 trials in the acquisition phase. The histogram shows the mean percentage of non-reinforced choices made during 45 seconds for the rewarded stimulus (A+; black bar) and the punished stimulus (B-; grey bar) in the test.* ***B)*** *2^nd^ phase of the reversal learning protocol. The curve shows the mean percentage of correct choices of the group for the rewarded stimulus (B+) during the 10 trials of the acquisition. The histogram shows the mean percentage of choices of the group made during 45 seconds in the non-reinforced test for the correct (B+, black bar) and incorrect stimulus (A-, grey bar) and* ***C)*** *Negative patterning. The curve shows the mean percentage of choices of the group for the incorrect mixture stimulus (CD-) throughout the 20 trials of the acquisition. As the test performances of the two single stimuli (C+ and D+) did not differ from each other, they were pooled for analysis. The histogram shows the mean percentage of choices pooled for the correct two single stimuli (C+/D+) and the incorrect mixture stimulus made during 45 seconds in the non-reinforced test.* **** p≤ 0.001*

***Fig. S7*** *Group acquisition curves and group test performances of reversal learning (A+B) and negative patterning (C) protocols in experiment 3 (N = 140).* ***A)*** *1^st^ phase of the reversal learning protocol. The curve shows the percentage of bees (N = 140) that responded with an extension of the proboscis (% PER) to either the correct stimulus (A+; black line) or the incorrect stimulus (B-; grey line) during the 8 trials each of the acquisition phase. The histogram shows the percentage of bees responding with an extension of the proboscis to the correct (A+; black bar) and incorrect stimulus (B-; grey bar) in the non-reinforced test.* ***B)*** *2^nd^ phase of the reversal learning protocol. The curves show the percentage of bees (N = 140) that responded with a PER to either the correct stimulus (B+; black line) or the incorrect stimulus (A-; grey line) during the 8 trials each of the acquisition. The histogram shows the percentage of bees responding with a PER to the correct stimulus (B+, black bar) and the incorrect stimulus (A-, grey bar) in the non-reinforced test.* ***C)*** *Negative patterning. The curve shows the percentage of bees responding with a PER to either the two correct single stimuli (C+/D+; black line) or their incorrect mixture (CD-, grey line) during the 8 blocks of trials of the acquisition. Each block consisted of one presentation of the two single stimuli and two presentations of the mixture. The histogram shows the percentage of bees responding with a PER to the two correct single stimuli (C+/D+, black bar) and to their incorrect mixture (CD; grey bar). The responses to the two single stimuli were not different in the acquisition, nor in the test and thus pooled for analysis. *** p≤ 0.001*

**

***Fig. S8 Relative test performances of experiment 3: Visual learning experiments with restrained bees.*** *The bees were assigned to groups, depending on their performances in the test. A ‘correct response’ was defined as being fully correct in the test (1^st^ phase of reversal learning, 1^st^ RL: Response to A and not to B; 2^nd^ phase of reversal learning, 2^nd^ RL: Response to B and not to A; Negative patterning, NP: Response to C and D but not to CD). A bee was scored as ‘incorrect response’ if it responded to the incorrect stimulus alone or to both stimuli and as ‘no response’ if it responded to none of the stimuli despite showing an intact PER. Relative test performances of the bees in the* ***A)*** *2^nd^ phase of reversal learning depending on their test performance in the 1^st^ phase of reversal learning.* ***B)*** *negative patterning depending on their test performance in the 1^st^ phase of reversal learning and* ***C)*** *negative patterning depending on their test performance in the 2^nd^ phase of reversal learning*

***Fig. S9*** *Group acquisition curves and group test performances of reversal learning (A+B) and negative patterning (C) protocols in experiment 4.* ***A)*** *1^st^ phase of reversal learning. The curve shows the percentage of bees (N = 89) that responded with an extension of the proboscis (% PER) to either the correct stimulus (A+; black line) or the incorrect stimulus (B-; grey line) during the 5 trials each in the acquisition phase. The histogram shows the percentage of bees responding with a PER to the correct (A+; black bar) and incorrect stimulus (B-; grey bar) in the non-reinforced test.* ***B)*** *2^nd^ phase of reversal learning. The curve shows the percentage of bees (N = 89) that responded with a PER to either the correct stimulus (B+; black line) and the incorrect stimulus (A-; grey line) during the 5 trials each of the acquisition. The histogram shows the percentage of bees responding with a PER to the correct stimulus (B+, black bar) and the incorrect stimulus (A-, grey bar) in the non-reinforced test.* ***C)*** *Negative patterning paradigm. The curve shows the percentage of bees responding with a PER to either the two correct single stimuli (C+/D+; black line) and their incorrect mixture (CD-; grey line) during the 5 blocks of trials of the acquisition. Each block consisted of one presentation with the two single stimuli and two presentations of the mixture. The histogram shows the percentage of bees showing a PER to the two correct single stimuli (C+/D+; black bar) and to their incorrect mixture (CD; grey bar) in the non-reinforced test. The responses to the two single stimuli were not different and thus pooled for analysis. *** p≤ 0.001*

***Fig. S10*** *Relative test performances of experiment 4: Olfactory learning experiments with restrained bees. The bees were assigned to groups, depending on their performances in the test. A ‘correct response’ was defined as being fully correct in the test (1^st^ phase of reversal learning, 1^st^ RL: Response to A and not to B; 2^nd^ phase of reversal learning, 2^nd^ RL: Response to B and not to A; Negative patterning, NP: Response to C and D but not to CD). A bee was scored as ‘incorrect response’ if it responded to the incorrect stimulus alone or to both stimuli and as ‘no response’ if it responded to none of the stimuli despite showing an intact PER. Relative test performances of the bees in the* ***A)*** *2^nd^ phase of reversal learning depending on their test performance in the 1^st^ phase of reversal learning.* ***B)*** *the negative patterning paradigm depending on their test performance in the 1^st^ phase of reversal learning and* ***C)*** *the negative patterning paradigm depending on their test performance in the 2^nd^ phase of reversal learning.*

**Statistical tables**

***Table S1*** *GLMM analysis of the bees’ performance in the acquisition of the 1^st^ phase of reversal learning in experiment 1. The model with the best fit is highlighted in bold. The p-value indicates the comparison of the concerning model with the model including one level of higher complexity.*

| **Models** | **df** | **AIC** | **Log-Lik** | **χ^2^** | **p(>χ^2^)** |
| --- | --- | --- | --- | --- | --- |
| model1: response ~ trial*order*group_RL + (1\|subject) | 9 | 1279.8 | -630.88 | - | - |
| model2: response ~ trial*order + group_RL + (1\|subject) | 6 | 1277.0 | -632.51 | 3.27 | 0.35 |
| model3: response ~ trial + order*group_RL + (1\|subject) | 6 | 1278.6 | -633.32 | 4.88 | 0.18 |
| model4: response ~ order + trial * group_RL + (1 \| subject) | 6 | 1278.6 | -633.3 | 4.84 | 0.18 |
| model5: response ~ trial + order + group_RL + (1 \| subject) | 5 | 1276.6 | -633.32 | 4.88 | 0.30 |
| model6: response ~ trial + group_RL + (1 \| subject) | 4 | 1277.9 | -634.96 | 3.29 | 0.07 |
| **model7: response ~ trial + (1 \| subject)** | **3** | **1275.9** | **-634.97** | **0.01** | **0.91** |
| model8: response ~ (1 \| subject) | 2 | 1301.2 | -648.59 | 27.24 | < 0.0001 |

***Table S2*** *GLMM analysis of the bees’ performance in the acquisition of the 2^nd^ phase of reversal learning in experiment 1. The model with the best fit is highlighted in bold. The p-value indicates the comparison of the concerning model with the model including one level of higher complexity.*

| **Models** | **df** | **AIC** | **Log-Lik** | **χ^2^** | **p(>χ^2^)** | |
| --- | --- | --- | --- | --- | --- | --- |
| model1: response ~ trial * order * group_RL + (1 \|subject) | 9 | 1337.1 | -659.56 | - | - |  |
| model2: response ~ trial * order + group_RL + (1 \|subject) | 6 | 1331.5 | -659.72 | 0.34 | 0.95 |  |
| model3: response ~ trial + order * group_RL + (1 \|subject) | 6 | 1331.9 | -659.95 | 0.78 | 0.85 |  |
| model4: response ~ order + trial * group_RL + (1 \|subject) | 6 | 1331.8 | -659.91 | 0.7 | 0.87 |  |
| model5: response ~ trial + order + group_RL + (1 \|subject) | 5 | 1329.9 | -659.95 | 0.78 | 0.94 |  |
| model6: response ~ trial + group_RL + (1 \|subject) | 4 | 1334.2 | -663.08 | 6.27 | 0.01 |  |
| **model7: response ~ trial + order + (1 \|subject)** | **4** | **1327.9** | **-659.97** | **0.04** | **0.845** |  |
| model8: response ~ order + (1 \|subject) | 3 | 1353.0 | -673.49 | 27.1 | < 0.0001 |  |

***Table S3*** *GLMM analysis of the bees’ performance in the acquisition of the negative patterning paradigm in experiment 1. The model with the best fit is highlighted in bold. The p-value indicates the comparison of the concerning model with the model including one level of higher complexity.*

| **Models** | **df** | **AIC** | **Log-Lik** | **χ^2^** | **p(>χ^2^)** |
| --- | --- | --- | --- | --- | --- |
| model1: response ~ trial*order*group_RL + (1\|subject) | 9 | 2322.1 | -1152.6 | - | - |
| model2: response ~ trial*order + group_RL + (1\|subject) | 6 | 2317.3 | -1152.6 | 1.15 | 0.76 |
| model3: response ~ trial + order*group_RL + (1\|subject) | 6 | 2316.8 | -1152.4 | 0.63 | 0.89 |
| model4: response ~ order + trial * group_RL + (1 \| subject) | 6 | 2316.9 | -1152.5 | 0.79 | 0.85 |
| model5: response ~ trial + order + group_RL + (1 \| subject) | 5 | 2315.3 | 2343.3 | 1.17 | 0.88 |
| model6: response ~ trial + group_RL + (1 \| subject) | 4 | 2313.4 | -1152.7 | 0.08 | 0.78 |
| **model7: response ~ trial + (1 \| subject)** | **3** | **2311.4** | **-1152.7** | **0.01** | **0.92** |
| model8: response ~ (1 \| subject) | 2 | 2374.0 | -1185.0 | 64.6 | < 0.0001 |

***Table S4*** *GLMM analysis of the bees’ performances in the non-reinforced tests of the 1^st^ phase of reversal learning in experiment 1. The model with the best fit is highlighted in bold. The p-value indicates the comparison of the concerning model with the model including one level of higher complexity.*

| **Models** | **df** | **AIC** | **Log-Lik** | **χ^2^** | **p(>χ^2^)** |
| --- | --- | --- | --- | --- | --- |
| model1: response ~ order*group_RL + (1 \| subject) | 5 | 785.87 | -387.94 | - | - |
| model2: response ~ order + group_RL + (1 \| subject) | 4 | 785.88 | -387.94 | 0.01 | 0.94 |
| model3: response ~ order + (1 \| subject) | 3 | 786.94 | -390.47 | 2.1 | 0.08 |
| **model4: response ~ (1 \| subject)** | **2** | **785.26** | **-390.63** | **0.32** | **0.57** |

***Table S5*** *GLMM analysis of the bees’ performances in the non-reinforced tests of the 2^nd^ phase of reversal learning in experiment 1. The model with the best fit is highlighted in bold. The p-value indicates the comparison of the concerning model with the model including one level of higher complexity.*

| **Models** | **df** | **AIC** | **Log-Lik** | **χ^2^** | **p(>χ^2^)** |
| --- | --- | --- | --- | --- | --- |
| model1: response ~ order*group_RL + (1 \| subject) | 5 | 903.77 | -446.88 | - | - |
| model2: response ~ order + group_RL + (1 \| subject) | 4 | 902.33 | -447.17 | 0.56 | 0.45 |
| model3: response ~ order + (1 \| subject) | 3 | 901.74 | -447.87 | 1.41 | 0.24 |
| **model4: response ~ (1 \| subject)** | **2** | **899.77** | **-447.89** | **0.04** | **0.85** |

***Table S6*** *GLMM analysis of the bees’ performances in the non-reinforced tests of the negative patterning paradigm in experiment 1. The model with the best fit is highlighted in bold. The p-value indicates the comparison of the concerning model with the model including one level of higher complexity.*

| **Models** | **df** | **AIC** | **Log-Lik** | **χ^2^** | **p(>χ^2^)** |
| --- | --- | --- | --- | --- | --- |
| model1: response ~ order*group_RL + (1 \| subject) | 5 | 1658.0 | -824.01 | - | - |
| model2: response ~ order + group_RL + (1 \| subject) | 4 | 1656.1 | -824.01 | 0.04 | 0.84 |
| model3: response ~ order + (1 \| subject) | 3 | 1654.2 | -824.11 | 0.15 | 0.70 |
| **model4: response ~ (1 \| subject)** | **2** | **1655.2** | **-825.59** | **2.96** | **0.09** |

***Table S7*** *GLMM analysis of the bees’ performance in the acquisition of the 1^st^ phase of reversal learning in experiment 2. The model with the best fit is highlighted in bold. The p-value indicates the comparison of the concerning model with the model including one level of higher complexity.*

| **Models** | **df** | **AIC** | **Log-Lik** | **χ^2^** | **p(>χ^2^)** |
| --- | --- | --- | --- | --- | --- |
| model1: response ~ trial*order*group_RL + (1\|subject) | 9 | 262.39 | -122.19 | - | - |
| model2: response ~ trial*order + group_RL + (1\|subject) | 6 | 256.79 | -122.40 | 0.41 | 0.82 |
| model3: response ~ trial + order*group_RL + (1\|subject) | 6 | 257.30 | -122.65 | 0.91 | 0.82 |
| model4: response ~ order + trial * group_RL + (1 \| subject) | 6 | 257.64 | -122.82 | 1.26 | 0.74 |
| model5: response ~ trial + order + group_RL + (1 \| subject) | 5 | 255.72 | -122.86 | 1.33 | 0.86 |
| model6: response ~ trial + group_RL + (1 \| subject) | 4 | 254.76 | -123.38 | 1.04 | 0.31 |
| **model7: response ~ trial + (1 \| subject)** | **3** | **253.38** | **-123.69** | **0.63** | **0.43** |
| model8: response ~ (1 \| subject) | 2 | 290.10 | -143.05 | 38.71 | < 0.0001 |

***Table S8. GLMM analysis of the bees’ performance in the acquisition of the 2^nd^ phase of reversal learning in experiment 2.*** *The model with the best fit is highlighted in bold. The p-value indicates the comparison of the concerning model with the model including one level of higher complexity.*

| **Models** | **df** | **AIC** | **Log-Lik** | **χ^2^** | **p(>χ^2^)** |
| --- | --- | --- | --- | --- | --- |
| model1: response ~ trial*order*group_RL + (1\|subject) | 9 | 268.77 | -125.39 | - | - |
| model2: response ~ trial*order + group_RL + (1\|subject) | 6 | 264.25 | -126.12 | 1.48 | 0.69 |
| model3: response ~ trial + order*group_RL + (1\|subject) | 6 | 264.06 | -126.03 | 1.29 | 0.73 |
| model4: response ~ order + trial * group_RL + (1 \| subject) | 6 | 264.44 | -126.22 | 1.67 | 0.64 |
| model5: response ~ trial + order + group_RL + (1 \| subject) | 5 | 262.63 | -126.31 | 1.86 | 0.76 |
| model6: response ~ trial + group_RL + (1 \| subject) | 4 | 262.26 | -127.13 | 1.63 | 0.20 |
| **model7: response ~ trial + (1 \| subject)** | **3** | **260.63** | **-127.32** | **0.37** | **0.54** |
| model8: response ~ (1 \| subject) | 2 | 303.75 | -149.87 | 45.11 | < 0.0001 |

***Table S9. GLMM analysis of the bees’ performance in the acquisition of the negative patterning paradigm in experiment 2.*** *The model with the best fit is highlighted in bold. The p-value indicates the comparison of the concerning model with the model including one level of higher complexity.*

| **Models** | **df** | **AIC** | **Log-Lik** | **χ^2^** | **p(>χ^2^)** |
| --- | --- | --- | --- | --- | --- |
| model1: response ~ trial*order*group_RL + (1\|subject) | 9 | 582.46 | -282.23 | - | - |
| model2: response ~ trial*order + group_RL + (1\|subject) | 6 | 578.16 | -283.08 | 1.70 | 0.64 |
| model3: response ~ trial + order*group_RL + (1\|subject) | 6 | 577.65 | -282.83 | 1.19 | 0.76 |
| model4: response ~ order + trial * group_RL + (1 \| subject) | 6 | 577.61 | -282.81 | 1.15 | 0.77 |
| model5: response ~ trial + order + group_RL + (1 \| subject) | 5 | 576.1 | -283.09 | 1.71 | 0.79 |
| model6: response ~ trial + group_RL + (1 \| subject) | 4 | 576.27 | -284.14 | 2.10 | 0.15 |
| **model7: response ~ trial + (1 \| subject)** | **3** | **576.50** | **-285.25** | **2.22** | **0.14** |
| model8: response ~ (1 \| subject) | 2 | 603.42 | -299.71 | 28.92 | < 0.0001 |

***Table S10. GLMM analysis of the bees’ performances in the non-reinforced tests of the 1^st^ phase of reversal learning in experiment 2.*** *The model with the best fit is highlighted in bold. The p-value indicates the comparison of the concerning model with the model including one level of higher complexity.*

| **Models** | **df** | **AIC** | **Log-Lik** | **χ^2^** | **p(>χ^2^)** |
| --- | --- | --- | --- | --- | --- |
| model1: response ~ order * group_RL + (1 \| subject) | 5 | 338.00 | -164.00 | - | - |
| model2: response ~ order + group_RL + (1 \| subject) | 4 | 336.26 | -164.13 | 0.26 | 0.61 |
| model3: response ~ order + (1 \| subject) | 3 | 334.44 | -164.22 | 0.17 | 0.68 |
| **model4: response ~ (1 \| subject)** | **2** | **334.47** | **-165.24** | **2.03** | **0.15** |

***Table S11. GLMM analysis of the bees’ performances in the non-reinforced tests of the 2^nd^ phase of reversal learning in experiment 2.*** *The model with the best fit is highlighted in bold. The p-value indicates the comparison of the concerning model with the model including one level of higher complexity.*

| **Models** | **df** | **AIC** | **Log-Lik** | **χ^2^** | **p(>χ^2^)** |
| --- | --- | --- | --- | --- | --- |
| model1: response ~ order * group_RL + (1 \| subject) | 5 | 394.87 | -192.43 | - | - |
| model2: response ~ order + group_RL + (1 \| subject) | 4 | 394.87 | -193.07 | 1.26 | 0.26 |
| model3: response ~ order + (1 \| subject) | 3 | 392.48 | -193.24 | 0.35 | 0.55 |
| **model4: response ~ (1 \| subject)** | **2** | **390.53** | **-193.26** | **0.05** | **0.83** |

***Table S12. GLMM analysis of the bees’ performances in the non-reinforced tests of the negative patterning paradigm in experiment 2****. The model with the best fit is highlighted in bold. The p-value indicates the comparison of the concerning model with the model including one level of higher complexity.*

| **Models** | **df** | **AIC** | **Log-Lik** | **χ^2^** | **p(>χ^2^)** |
| --- | --- | --- | --- | --- | --- |
| model1: response ~ order * group_RL + (1 \| subject) | 5 | 353.05 | -171.53 | - | - |
| model2: response ~ order + group_RL + (1 \| subject) | 4 | 352.08 | -172.04 | 1.03 | 0.31 |
| model3: response ~ order + (1 \| subject) | 3 | 351.08 | -172.54 | 1 | 0.32 |
| **model4: response ~ (1 \| subject)** | **2** | **349.11** | **-172.56** | **0.04** | **0.82** |

***Table S13. GLMM analysis of the bees’ performance in the acquisition of the 1^st^ phase of reversal learning paradigm in experiment 3.*** *The model with the best fit is highlighted in bold. The p-value indicates the comparison of the concerning model with the model including one level of higher complexity.*

| **Models** | **df** | **AIC** | **Log-Lik** | **χ^2^** | **p(>χ^2^)** |
| --- | --- | --- | --- | --- | --- |
| model1: response ~ trial * order * group_RL * CS + (1 \| subject) | 17 | 2376.9 | -1171.4 | - | - |
| model2: response ~ trial * order * group_RL + CS + (1 \| subject) | 10 | 2428.9 | -1204.5 | 66.04 | < 0.0001 |
| model3: response ~ trial * order * CS + group_RL + (1 \| subject) | 10 | 2388.4 | -1204.5 | 40.53 | < 0.0001 |
| model4: response ~ trial * group_RL * CS + order + (1 \| subject) | 10 | 2374.5 | -1177.3 | 0 | 1 |
| **model5: response ~ trial * group_RL*CS + (1 \| subject)** | **9** | **2372.5** | **-1177.3** | **0.12** | **0.73** |

***Table S14. GLMM analysis of the bees’ performance in the acquisition of the 2^nd^ phase of reversal learning in experiment 3.*** *The model with the best fit is highlighted in bold. The p-value indicates the comparison of the concerning model with the model including one level of higher complexity.*

| **Models** | **df** | **AIC** | **Log-Lik** | **χ^2^** | **p(>χ^2^)** |
| --- | --- | --- | --- | --- | --- |
| **model1: response ~ trial * order * group_RL * CS + (1 \| subject)** | **17** | **2396.1** | **-1181.0** | **-** | **-** |
| model2: response ~ trial * order * group_RL + CS + (1 \| subject) | 10 | 2546.6 | -1263.3 | 164.5 | < 0.0001 |
| model3: response ~ trial * order * CS + group_RL + (1 \| subject) | 10 | 2413.7 | -1196.9 | 31.69 | < 0.0001 |
| model4: response ~ trial * group_RL * CS + order + (1 \| subject) | 9 | 2546.6 | -1263.3 | 164.5 | < 0.0001 |

***Table S15. GLMM analysis of the bees’ performance in the acquisition of the negative patterning paradigm in experiment 3.*** *The model with the best fit is highlighted in bold. The p-value indicates the comparison of the concerning model with the model including one level of higher complexity.*

| **Models** | **df** | **AIC** | **Log-Lik** | **χ^2^** | **p(>χ^2^)** |
| --- | --- | --- | --- | --- | --- |
| model1: response ~ trial * order * group_RL * CS + (1 \| subject) | 25 | 4814.6 | -2382.2 | - | - |
| model2: response ~ trial * order * group_RL + CS + (1 \| subject) | 11 | 4975.1 | -2476.5 | 188.5 | < 0.0001 |
| **model3: response ~ trial * order * CS + group_RL + (1 \| subject)** | **14** | **4812.7** | **-2392.4** | **168.3** | **< 0.0001** |
| model4: response ~ trial * group_RL * CS + order + (1 \| subject) | 14 | 4846.2 | -2409.1 | 0 | 1 |
| model5: response ~ trial * group_RL*CS + (1 \| subject) | 13 | 4847.6 | -2410.8 | 3.38 | 0.07 |

***Table S16. GLMM analysis of the bees’ performances in the non-reinforced tests of the 1^st^ phase of reversal learning in experiment 3.*** *The model with the best fit is highlighted in bold. The p-value indicates the comparison of the concerning model with the model including one level of higher complexity.*

| **Models** | **df** | **AIC** | **Log-Lik** | **χ^2^** | **p(>χ^2^)** |
| --- | --- | --- | --- | --- | --- |
| model1: response ~ CS*order*group_RL + (1\|subject) | 9 | 168.10 | -75.05 | - | - |
| model2: response ~ CS*order + group_RL + (1\|subject) | 6 | 162.29 | -75.15 | 0.19 | 0.9 |
| model3: response ~ CS + order*group_RL + (1\|subject) | 6 | 162.10 | -75.05 | 0 | 1 |
| model4: response ~ order + CS* group_RL + (1 \| subject) | 6 | 162.29 | -75.15 | 0.19 | 0.9 |
| model5: response ~ CS + order + group_RL + (1 \| subject) | 5 | 160.29 | -75.15 | 0.19 | 0.9 |
| model6: response ~ CS + group_RL + (1 \| subject) | 4 | 158.34 | -75.17 | 0.04 | 0.8 |
| **model7: response ~ CS + (1 \| subject)** | **3** | **157.18** | **-75.59** | **0.84** | **0.3** |
| model8: response ~ (1 \| subject) | 2 | 263.76 | -129.88 | 108.6 | < 0.0001 |

***Table S17. GLMM analysis of the bees’ performances in the non-reinforced tests of the 2^nd^ phase of reversal learning in experiment 3.*** *The model with the best fit is highlighted in bold. The p-value indicates the comparison of the concerning model with the model including one level of higher complexity.*

| **Models** | **df** | **AIC** | **Log-Lik** | **χ^2^** | **p(>χ^2^)** |
| --- | --- | --- | --- | --- | --- |
| model1: response ~ CS*order*group_RL + (1\|subject) | 9 | 292.12 | -137.06 | - | - |
| model2: response ~ CS*order + group_RL + (1\|subject) | 6 | 290.32 | -139.16 | 4.20 | 0.24 |
| model3: response ~ CS + order*group_RL + (1\|subject) | 6 | 291.91 | -139.95 | 5.79 | 0.12 |
| model4: response ~ order + CS* group_RL + (1 \| subject) | 6 | 290.26 | -139.13 | 4.14 | 0.25 |
| model5: response ~ CS + order + group_RL + (1 \| subject) | 5 | 290.09 | -140.04 | 5.97 | 0.20 |
| model6: response ~ CS + group_RL + (1 \| subject) | 4 | 288.09 | -140.04 | 0.002 | 0.97 |
| **model7: response ~ CS + (1 \| subject)** | **3** | **288.09** | **-140.04** | **3.74** | **0.06** |
| model8: response ~ (1 \| subject) | 2 | 303.71 | -149.85 | 15.88 | < 0.0001 |

***Table S18. GLMM analysis of the bees’ performances in the non-reinforced tests of negative patterning in experiment 3.*** *The model with the best fit is highlighted in bold. The p-value indicates the comparison of the concerning model with the model including one level of higher complexity.*

| **Models** | **df** | **AIC** | **Log-Lik** | **χ^2^** | **p(>χ^2^)** |
| --- | --- | --- | --- | --- | --- |
| model1: response ~ CS*order*group_RL + (1\|subject) | 13 | 381.93 | -177.97 | - | - |
| model2: response ~ CS*order + group_RL + (1\|subject) | 8 | 372.92 | -178.46 | 0.99 | 0.96 |
| model3: response ~ CS + order*group_RL + (1\|subject) | 8 | 370.53 | -178.26 | 0.60 | 1 |
| model4: response ~ order + CS* group_RL + (1 \| subject) | 8 | 372.87 | -178.43 | 0.93 | 0.97 |
| model5: response ~ CS + order + group_RL + (1 \| subject) | 6 | 369.10 | -178.55 | 1.16 | 0.99 |
| model6: response ~ CS + group_RL + (1 \| subject) | 5 | 367.12 | -178.56 | 0.03 | 0.86 |
| **model7: response ~ CS + (1 \| subject)** | **4** | **366.70** | **-179.35** | **1.58** | **0.21** |
| model8: response ~ (1 \| subject) | 3 | 436.93 | -216.47 | 74.23 | < 0.0001 |

***Table S19. GLMM analysis of the bees’ performance in the acquisition of the 1^st^ phase of reversal learning in experiment 4.*** *The model with the best fit is highlighted in bold. The p-value indicates the comparison of the concerning model with the model including one level of higher complexity.*

| **Models** | **df** | **AIC** | **Log-Lik** | **χ^2^** | **p(>χ^2^)** |
| --- | --- | --- | --- | --- | --- |
| model1: response ~ trial * order * group_RL * CS + (1 \| subject) | 57 | 2842.2 | -1364.1 | - | - |
| model2: response ~ trial * order * group_RL + CS + (1 \| subject) | 15 | 3032.3 | -1501.2 | 274.1 | < 0.0001 |
| **model3: response ~ trial * order * CS + group_RL + (1 \| subject)** | **30** | **2830.2** | **-1385.1** | **232.1** | **< 0.0001** |
| model4: response ~ trial * group_RL * CS + order + (1 \| subject) | 30 | 2902.9 | -1421.5 | **0** | **1** |
| model5: response ~ trial * group_RL*CS + (1 \| subject) | 29 | 2906.3 | -1424.1 | 5.34 | 0.03 |

***Table S20. GLMM analysis of the bees’ performance in the acquisition of the 2^nd^ phase of reversal learning in experiment 4.*** *The model with the best fit is highlighted in bold. The p-value indicates the comparison of the concerning model with the model including one level of higher complexity.*

| **Models** | **df** | **AIC** | **Log-Lik** | **χ^2^** | **p(>χ^2^)** |
| --- | --- | --- | --- | --- | --- |
| model1: response ~ trial * order * group_RL * CS + (1 \| subject) | 57 | 2851.5 | -1368.8 | **-** | **-** |
| model2: response ~ trial * order * group_RL + CS + (1 \| subject) | 15 | 3059.3 | -1514.7 | 291.8 | < 0.0001 |
| **model3: response ~ trial * order * CS + group_RL + (1 \| subject)** | **30** | **2849.4** | **-1394.7** | **239.9** | **< 0.0001** |
| model4: response ~ trial * group_RL * CS + order + (1 \| subject) | 30 | 2921.1 | -1430.6 | 0 | 1 |
| model5: response ~ trial * group_RL*CS + (1 \| subject) | 12 | 2923.0 | -1432.5 | 3.83 | 0.06 |

***Table S21. GLMM analysis of the bees’ performance in the acquisition of the negative patterning paradigm in experiment 4****. The model with the best fit is highlighted in bold. The p-value indicates the comparison of the concerning model with the model including one level of higher complexity.*

| **Models** | **df** | **AIC** | **Log-Lik** | **χ^2^** | **p(>χ^2^)** |
| --- | --- | --- | --- | --- | --- |
| **model1: response ~ trial * order * group_RL * CS + (1 \| subject)** | **57** | **3053.0** | **-1469.5** | **-** | **-** |
| model2: response ~ trial * order * group_RL + CS + (1 \| subject) | 15 | 3262.9 | -1616.5 | 293.9 | < 0.0001 |
| model3: response ~ trial * order * CS + group_RL + (1 \| subject) | 30 | 3235.6 | -1496.9 | 239 | < 0.0001 |
| model4: response ~ trial * group_RL * CS + order + (1 \| subject) | 30 | 3133.1 | -1536.6 | 0 | 1 |
| **model5: response ~ trial * group_RL*CS + (1 \| subject)** | **29** | **3132.7** | **-1537.4** | **1.6** | **0.21** |

***Table S22. GLMM analysis of the bees’ performances in the non-reinforced tests of the 1^st^ phase of reversal learning in experiment 4.*** *The model with the best fit is highlighted in bold. The p-value indicates the comparison of the concerning model with the model including one level of higher complexity.*

| **Models** | **df** | **AIC** | **Log-Lik** | **χ^2^** | **p(>χ^2^)** |
| --- | --- | --- | --- | --- | --- |
| model1: response ~ CS*order*group_RL + (1\|subject) | 9 | 203.74 | -92.87 | - | - |
| model2: response ~ CS*order + group_RL + (1\|subject) | 6 | 205.99 | -96.99 | 8.25 | 0.04 |
| model3: response ~ CS + order*group_RL + (1\|subject) | 6 | 202.12 | -95.06 | 4.38 | 0.22 |
| model4: response ~ order + CS* group_RL + (1 \| subject) | 6 | 203.13 | -98.56 | 3.43 | 0.33 |
| model5: response ~ CS + order + group_RL + (1 \| subject) | 5 | 204.04 | -97.02 | 8.31 | 0.08 |
| model6: response ~ CS + group_RL + (1 \| subject) | 4 | 205.11 | -98.56 | 3.07 | 0.08 |
| **model7: response ~ CS + (1 \| subject)** | **3** | **201.17** | **-94.59** | **0.01** | **0.9** |
| model8: response ~ (1 \| subject) | 2 | 244.23 | -120.11 | 43.10 | < 0.0001 |

***Table S23. GLMM analysis of the bees’ performances in the non-reinforced tests of the 2^nd^ phase of reversal learning in experiment 4.*** *The model with the best fit is highlighted in bold. The p-value indicates the comparison of the concerning model with the model including one level of higher complexity.*

| **Models** | **df** | **AIC** | **Log-Lik** | **χ^2^** | **p(>χ^2^)** |
| --- | --- | --- | --- | --- | --- |
| model1: response ~ CS*order*group_RL + (1\|subject) | 9 | 234.89 | -108.44 | - | - |
| model2: response ~ CS*order + group_RL + (1\|subject) | 6 | 230.56 | -109.28 | 1.67 | 0.64 |
| model3: response ~ CS + order*group_RL + (1\|subject) | 6 | 231.42 | -109.71 | 2.53 | 0.47 |
| model4: response ~ order + CS* group_RL + (1 \| subject) | 6 | 231.89 | -109.95 | 3.01 | 0.39 |
| model5: response ~ CS + order + group_RL + (1 \| subject) | 5 | 230.05 | -110.03 | 3.16 | 0.53 |
| model6: response ~ CS + group_RL + (1 \| subject) | 4 | 228.16 | -110.08 | 0.10 | 0.75 |
| **model7: response ~ CS + (1 \| subject)** | **3** | **226.92** | **-110.46** | **0.76** | **0.38** |
| model8: response ~ (1 \| subject) | 2 | 243.76 | -119.88 | 18.85 | < 0.0001 |

***Table S24. GLMM analysis of the bees’ performances in the non-reinforced tests of negative patterning in experiment 4.*** *The model with the best fit is highlighted in bold. The p-value indicates the comparison of the concerning model with the model including one level of higher complexity.*

| **Models** | **df** | **AIC** | **Log-Lik** | **χ^2^** | **p(>χ^2^)** |
| --- | --- | --- | --- | --- | --- |
| model1: response ~ CS*order*group_RL + (1\|subject) | 13 | 271.42 | -122.71 | - | - |
| model2: response ~ CS*order + group_RL + (1\|subject) | 8 | 264.29 | -124.14 | 2.87 | 0.72 |
| model3: response ~ CS + order*group_RL + (1\|subject) | 8 | 257.10 | -121.55 | 0 | 1 |
| model4: response ~ order + CS* group_RL + (1 \| subject) | 8 | 263.99 | -124.00 | 2.57 | 0.77 |
| model5: response ~ CS + order + group_RL + (1 \| subject) | 6 | 261.67 | -124.83 | 4.25 | 0.75 |
| model6: response ~ CS + group_RL + (1 \| subject) | 4 | 260.18 | -125.09 | 0.51 | 0.47 |
| **model7: response ~ CS + (1 \| subject)** | **3** | **258.31** | **-125.16** | **0.13** | **0.72** |
| model8: response ~ (1 \| subject) | 2 | 346.93 | -171.47 | 92.62 | < 0.0001 |

**References**

Bates D, Mächler M, Bolker B, Walker S (2015) Fitting linear mixed-effects models using lme4. J Stat Softw 67:. https://doi.org/10.18637/jss.v067.i01

Burnham KP, Anderson DR (1998) A practical information-theoretic approach. In: Model selection and inference. Springer, New York, NY, pp 75–117

Chittka L (1992) The colour hexagon: a chromaticity diagram based on photoreceptor excitations as a generalized representation of colour opponency. J Comp Physiol A 170:533–543. https://doi.org/10.1007/BF00199331

Fox J, Weisberg S (2019) An R companion to applied regression, third edition. Sage, Thousand Oaks CA

Judd DB, MacAdam DL, Wyszecki G, et al (1964) Spectral distribution of typical daylight as a function of correlated color temperature. J Opt Soc Am 54:1031–1040. https://doi.org/10.1364/JOSA.54.001031

Panchal G, Ganatra A, Kosta YP, Panchal D (2010) Searching most efficient neural network architecture using Akaike’s information criterion (AIC). Int J Comput Appl 1:41–44

Peitsch D, Fietz A, Hertel H, et al (1992) The spectral input systems of hymenopteran insects and their receptor-based colour vision. J Comp Physiol A 170:23–40. https://doi.org/10.1007/BF00190398

R Core Team (2022) R: A language and environment for statistical computing. R Found Stat Comput Vienna, Austria
